# Simultaneity in binary outcome models with an application to employment for couples

**DOI:** 10.1007/s00181-023-02417-7

**Published:** 2023-05-04

**Authors:** Bo E. Honoré, Luojia Hu, Ekaterini Kyriazidou, Martin Weidner

**Affiliations:** 1 Princeton University, Princeton, USA; 2 Federal Reserve Bank of Chicago, Chicago, USA; 3 New York University Abu Dhabi, Abu Dhabi, UAE; 4 University of Oxford, Oxford, UK

**Keywords:** Simultaneity, Binary response, Fixed effects, Moment conditions, Employment, C01, C33, C35, E24

## Abstract

Two of Peter Schmidt’s many contributions to econometrics have been to introduce a simultaneous logit model for bivariate binary outcomes and to study estimation of dynamic linear fixed effects panel data models using short panels. In this paper, we study a dynamic panel data version of the bivariate model introduced in Schmidt and Strauss (Econometrica 43:745–755, 1975) that allows for lagged dependent variables and fixed effects as in Ahn and Schmidt (J Econom 68:5–27, 1995). We combine a conditional likelihood approach with a method of moments approach to obtain an estimation strategy for the resulting model. We apply this estimation strategy to a simple model for the intra-household relationship in employment. Our main conclusion is that the within-household dependence in employment differs significantly by the ethnicity composition of the couple even after one allows for unobserved household specific heterogeneity.

## Introduction

A large recent literature has been concerned with econometric models in which binary outcomes interact with each other. The papers by Bresnahan and Reiss (1991) and Tamer (2003) are early examples of this. In those papers, the dependence is due to strategic interactions between economic agents. This literature was predated by Schmidt and Strauss (1975) who proposed a reduced form statistical model that has the feature that the conditional distribution of each binary variable depends on the outcome of the other.

At the same time, a large econometric literature has been concerned with estimation of linear panel data models with fixed effects and lagged dependent variables. This literature dates back to Nickell (1981) and Anderson and Hsiao (1982). The paper by Ahn and Schmidt (1995) is an important contribution to this literature.

This paper combines insights from these literatures by illustrating how the simultaneous binary outcome model in Schmidt and Strauss (1975) can be modified to allow for panel data with individual specific fixed effects and lagged dependent variables. The main contribution of the paper is to develop a toolbox of estimation procedures that can be used to estimate the resulting models.

Methodologically, the paper fits into the literature that is concerned with estimation of standard nonlinear panel data models with fixed effects using short panels. This literature has a long history in econometrics. The main problem to be solved is that treating the fixed effects as parameters to be estimated will typically lead to inconsistent estimation of all the model parameters. The literature has developed a number of methods to deal with this. One approach for parametric models is to try to construct a non-trivial sufficient statistic for the fixed effect. If such a sufficient statistic exists, then conditional maximum likelihood (conditional on this sufficient statistic) can typically be used to estimate the parameters of the model. This approach was, for example, taken by Rasch (1960) and Hausman et al. (1984) for the logit model and the Poisson regression model, respectively. Manski (1987) proposed a conditional maximum score estimator for the semiparametric binary response model with fixed effects, which can be thought of as a generalization of the conditional maximum likelihood approach. Honoré and Kyriazidou (2000) adapted both the conditional maximum likelihood and the conditional maximum score methods to binary outcome models with lagged dependent variables and fixed effects. A second strand of the literature has studied specific semiparametric models and has been able to find moment conditions which do not depend on the fixed effects, and which can therefore be used to estimate the model parameters via generalized method of moments. See for example, Honoré (1992), Chamberlain (1992), Kyriazidou (1997), Wooldridge (1997) , Kyriazidou (2001) and Hu (2002). More recently, Johnson (2004), Kitazawa (2013), Honoré and Weidner (2022) and Honoré et al. (2021) and Davezies et al. (2022) have derived moment conditions for parametric logit-type models with fixed effects, for which the conditional likelihood approach cannot be applied.

In this paper, we study estimation of a dynamic fixed effects panel data version of the Schmidt–Strauss model. It turns out that although the conditional likelihood approach can be applied to identify and estimate some of the parameters of the model, it does not identify the key parameter that captures the dependence between the binary outcomes. On the other hand, it turns out that one can construct moment conditions that do depend on this parameter, which can therefore be estimated by generalized method of moments.

As an empirical illustration of the models and methods studied in this paper, we investigate the joint determination of husbands’ and wives’ employment. In this context, it is natural to allow for the possibility that the outcome for each spouse is related to the outcome of the other, which makes it natural to consider the Schmidt–Strauss framework. The specific empirical question is how the parameter that captures the dependence between outcomes for husbands and wives differs by the ethnicity of the couple, and whether it varies over time. Since there is likely persistence in employment, and that some of this persistence might be due to heterogeneity as opposed to true state dependence, it is therefore natural to study this question using dynamic panel data versions of the model proposed by Schmidt and Strauss (1975).

The paper is organized as follows: In Sect. 2, we present the Schmidt and Strauss (1975) model. In Sect. 3, we discuss the data. Section 4 presents simple evidence for the intra-household dependence in couples ’ employment by ethnicity. Section 5 develops and discusses a conditional likelihood approach for estimating a version of the Schmidt and Strauss model that incorporates lagged dependent variables as well as fixed effects. Section 6 discusses how the method of moments approach of Honoré and Weidner (2022) can be used to identify the dependence parameter. In Sect. 7, we compare the fixed effects approach to a correlated random effects approach in the spirit of Wooldridge (2005). Section 8 concludes. The Appendix provides moment conditions for a special case of the model in Sect. 6.

## The Schmidt–Strauss model

Schmidt and Strauss (1975) proposed a cross-sectional simultaneous equations logit model in which two binary variables, $y_{1,i}$ and $y_{2,i}$, for a unit *i* are each distributed according to a logit model conditional on the other variable and on a set of explanatory variables

1 $$\begin{aligned} P\left( \left. y_{1,i}=1\right| y_{2,i},x_{1,i},x_{2,i}\right)= & {} \varLambda \left( x_{1,i}^{\prime }\beta _{1}+\rho y_{2,i}\right) , \\ P\left( \left. y_{2,i}=1\right| y_{1,i},x_{1,i},x_{2,i}\right)= & {} \varLambda \left( x_{2,i}^{\prime }\beta _{2}+\rho y_{1,i}\right) . \nonumber \end{aligned}$$

Here $$x_{1,i}$$ and $$x_{2,i}$$ are vectors of explanatory variables, $$\beta _1$$ , $$\beta _2$$ and $$\rho $$ are parameters to be estimated, and $$\varLambda \left( \cdot \right) $$ is the logistic cumulative distribution function. The parameter $$\rho $$ captures the dependence between $$y_{1,i}$$ and $$y_{2,i}$$. Schmidt and Strauss (1975) show that this model cannot be generalized to allow for different values for $$\rho $$ in the distribution of $$y_{1,i}$$ given $$y_{2,i}$$ and in the distribution of $$y_{2,i}$$ given $$y_{1,i}$$. In this sense, $$\rho $$ resembles the covariance between two random variables. When the parameter $$\rho $$ is positive (negative), the probability that $$ y_{1,i}$$ equals one is higher (lower) conditional on $$y_{2,i}$$ being one than conditional on $$y_{2,i}$$ being zero. The same holds for the probability that $$y_{2,i}$$ is one conditional on $$y_{1,i}$$. Holding the explanatory variables fixed, a positive (negative) $$\rho $$ therefore corresponds to a positive (negative) statistical association between $$y_{1,i}$$ and $$y_{2,i}$$.

The simultaneous logit model of Schmidt and Strauss (1975) has been applied in a variety of cross-sectional studies and in various fields such as labor economics (for example, by Lehrer and Stokes (1985) to study the determinant of different aspects of a chosen occupation), urban economics (for example, by Boehm (1981) to study the effects of various variables on the choice to own or rent and on expected future mobility), health economics (for example, by Akin et al. (1981) to study the use of different kinds of health services, and by Wang and Rosenman (2007) to study the need for health insurance on one hand and actual purchase of health insurance on the other), transportation (for example, by Ye et al. (2007) to study the relationship between mode of transportation and trip chaining), political economy (for example, by Kau et al. (1982) to study the interactions between congressional voting, campaign contributions and electorial margins), and demography (for example, by Koo and Janowitz (1983) to study the relationship between the probability of dissolving a marriage and of having a child).

The conditional probabilities in Eq. (1) emerge from a statistical model in which $y_{1,i}$ and $y_{2,i}$ have the joint probability distribution

2 $$\begin{aligned}&P\left( \left. y_{1,i}=c_{1},y_{2,i}=c_{2}\right| x_{1,i},x_{2,i}\right) \\&\quad =\frac{\exp \left( c_{1}x_{1,i}^{\prime }\beta _{1}+c_{2}x_{2,i}^{\prime }\beta _{2}+c_{1}c_{2}\rho \right) }{1+\exp \left( x_{1,i}^{\prime }\beta _{1}\right) +\exp (x_{2,i}^{\prime }\beta _{2})+\exp \left( x_{1,i}^{\prime }\beta _{1}+x_{2,i}^{\prime }\beta _{2}+\rho \right) }. \nonumber \end{aligned}$$

Another way to see that $\rho$ measures the dependence between $y_{1,i}$ and $y_{2,i}$ in Eq. (2), is to note that

3 $$\begin{aligned} \rho= & {} \log \left( P\left( \left. y_{1,i}=1,y_{2,i}=1\right| x_{1,i},x_{2,i}\right) \right) \nonumber \\{} & {} +\log \left( P\left( \left. y_{1,i}=0,y_{2,i}=0\right| x_{1,i},x_{2,i}\right) \right) \\{} & {} -\log \left( P\left( \left. y_{1,i}=0,y_{2,i}=1\right| x_{1,i},x_{2,i}\right) \right) \nonumber \\{} & {} -\log \left( P\left( \left. y_{1,i}=1,y_{2,i}=0\right| x_{1,i},x_{2,i}\right) \right) . \nonumber \end{aligned}$$

**Fig. 1:**
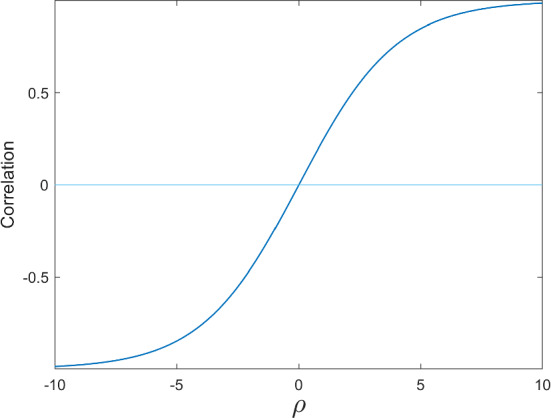
The Relationship between $\rho$ and the Correlation Coefficient. The figure shows the correlation between two Bernoulli random variables from the model in Eq. (2), each with probability of success equal to $\frac{1}{2}$ as a function of the parameter $\rho$

Therefore, $\log \left( P\left( \left. y_{1,i}=c_{1},y_{2,i}=c_{2}\right| x_{1,i},x_{2,i}\right) \right)$ is supermodular or submodular depending on whether $\rho >0$ or $\rho <0$. To understand how the magnitude of $\rho$, as opposed to its sign, translates into other measures of dependence, one can consider the following thought experiment: Suppose that, for a given $\rho$, $\beta _{1}$ and $\beta _{2}$ above are chosen such that $y_{1,i}$ and $y_{2,i}$ are Bernoulli, each with^1^ probability of success equal to 0.5. The correlation between $y_{1,i}$ and $y_{2,i}$ then relates to $\rho$ as depicted in Fig. 1.

Below, we apply the model of Schmidt and Strauss (1975) (and its panel data extensions) to an empirical study of husbands’ and wives’ employment status. In this context, *i* denotes the identity of the household, and $y_{1,i}$ and $y_{2,i}$ will denote the employment status of the wife and the husband, respectively. The next section introduces the data.

## Data

For the analysis in this paper, we use the Current Population Survey (CPS) Basic Monthly micro data from the 40 years between January of 1982 and December of 2021. The data are sourced from https://www.ipums.org/ (Flood et al. 2021). The monthly CPS has a panel design. Households are interviewed for four consecutive months, then not interviewed for eight months, and finally interviewed for four more consecutive months. We identify households with one head of household and one married or unmarried partner (of the head). The data consist of these heads and partners provided that they are of different sex and are both between the age of 25 and 65 (inclusive).^2^ Below, we sometimes refer to the partners as husbands and wives or as spouses although they are not always legally married. Since our ultimate goal is to investigate the dynamics of the employment status and a number of missing observations are missing in the last four months, we restrict the sample to the first four interview months, and we only use households who are in the sample in all of those 4 months.

We define four race/ethnicity groups: White, Black, Hispanic, and Other. Below we interchangeably refer to these groups as “race,”“ethnicity” or “race/ethnicity”. The couples are then grouped into five groups based on the race/ethnicity of the two partners: White, Black, Hispanic, Other, and Mixed Race. For example, White will refer to a couple, where both spouses are White, and “Mixed” will refer to a couple where the wife and husband have different ethnicity. We refer to these groups as the “ethnicity mix” (or sometimes just the “ethnicity”) of the couple.

Table 1 presents summary statistics for the variables used in this paper. The first is a dummy variable for working defined as the employment status being “At work”. The remaining variables are age in years, a dummy variable for the presence of children under the age of 5, a dummy variable for any children, and dummy variables for three education levels: high school or less, some college and college degree or more. Note that we report the number of individuals. Since this is a balanced panel with four time periods, the number of observations is larger than the number of individuals by a factor of four.

**Table 1:** Summary statistics by household ethnicity

	Women					
	All	Whites	Blacks	Hispanics	Other	Mixed
Working	0.64	0.65	0.67	0.52	0.62	0.67
Age	43.35	43.80	43.41	40.61	41.92	41.26
Kids < 5	0.19	0.18	0.18	0.28	0.25	0.23
Kids	0.65	0.63	0.69	0.81	0.77	0.65
HS or less	0.50	0.49	0.53	0.73	0.41	0.39
Some college	0.23	0.24	0.26	0.16	0.18	0.28
College+	0.27	0.28	0.21	0.10	0.42	0.33
No. individuals	1,002,489	783,312	54,342	63,999	39,765	61,071

	Men					
	All	Whites	Blacks	Hispanics	Other	Mixed
Working	0.83	0.84	0.76	0.83	0.82	0.84
Age	45.53	45.93	45.88	42.81	44.76	43.52
Kids < 5	0.19	0.18	0.18	0.28	0.25	0.23
Kids	0.65	0.63	0.69	0.81	0.77	0.65
HS or less	0.50	0.48	0.60	0.75	0.38	0.39
Some college	0.22	0.22	0.23	0.15	0.17	0.28
College+	0.29	0.30	0.17	0.10	0.44	0.33
No. individuals	1,002,489	783,312	54,342	63,999	39,765	61,071

The table shows averages by the ethnicity of the couple for the variables used in this paper. The data are from IPUMS CPS and cover a balanced panel of couples where each individual’s age is between 25 and 65. The data cover the period between 1982 and 2021

## Model and simple evidence

### Summary statistics

We start by presenting summary statistics for the joint probability of working by ethnicity. The first panel of Table 2 is for the whole sample, while the next two panels are for the subsamples of couples without children and with children. Our main takeaway from this table is that there is a large difference in these probabilities across the ethnicities, with Hispanic-Hispanic couples looking quite different from the others.

**Table 2:** Joint probabilities of employment by household ethnicity

		White		Black		Hispanic		Other		Mixed	
		Husband		Husband		Husband		Husband		Husband	
		No	Yes	No	Yes	No	Yes	No	Yes	No	Yes
		*All*									
Wife	No	0.087	0.260	0.114	0.217	0.096	0.388	0.088	0.294	0.074	0.258
	Yes	0.076	0.578	0.129	0.540	0.071	0.444	0.087	0.531	0.091	0.577
		*Without children*									
Wife	No	0.096	0.231	0.124	0.202	0.106	0.342	0.093	0.255	0.081	0.226
	Yes	0.084	0.589	0.138	0.537	0.080	0.471	0.096	0.557	0.100	0.593
		*With children*									
Wife	No	0.044	0.389	0.072	0.283	0.071	0.508	0.073	0.412	0.053	0.364
	Yes	0.039	0.528	0.090	0.555	0.048	0.373	0.062	0.453	0.059	0.523

The table shows the fraction of couples in each group that report each combination of working and not working. The data are from IPUMS CPS and cover a balanced panel of couples where each individual’s age is between 25 and 65. The data cover the period between 1982 and 2021

**Fig. 2:**
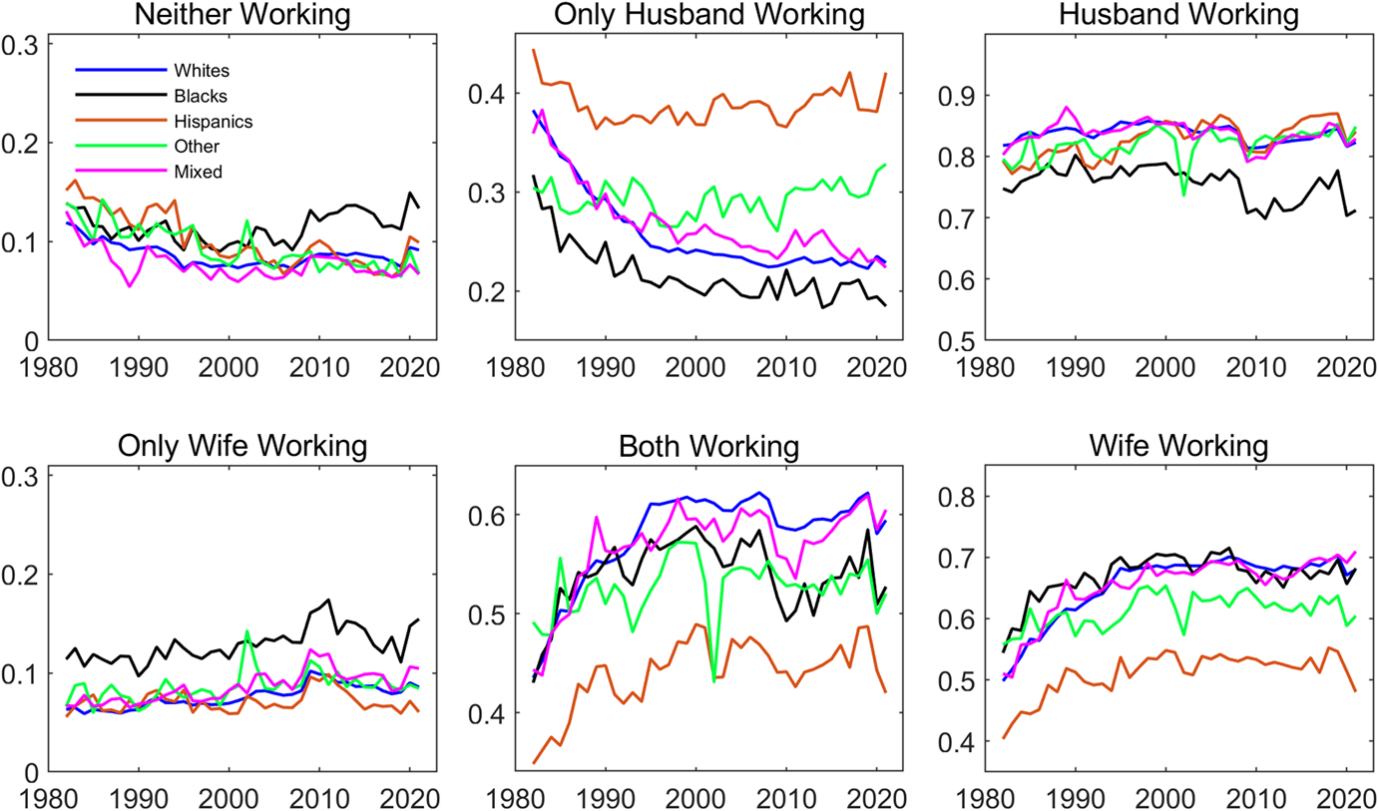
Probability Distributions of Employment over Time by Household Ethnicity. The data are from IPUMS CPS and cover a balanced panel of couples where each individual’s age is between 25 and 65. The data cover the period between 1982 and 2021

Table 2 aggregates the data for all years. In Fig. 2 we plot the joint probability of working over time for each ethnicity. These are depicted in the four leftmost plots. The two plots to the right are the marginal probabilities of working for the husbands and wives. Again, the main takeaway is that there are interesting differences across ethnicities, with Hispanics and, to a lesser extent, Blacks standing out. In terms of the evolution of the probabilities over time, the most distinct feature is the increase in the employment of women in the first part of the sample. This is seen in the marginal probabilities as well as the joint probabilities. It is also interesting that the 2008 recession had a large impact on the employment of men, but almost no effect for the women.

**Fig. 3:**
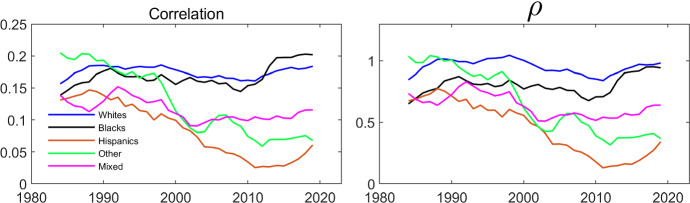
Evolution of Intra-Household Employment Dependence over Time by Household Ethnicity. The data are from IPUMS CPS and cover a balanced panel of couples where each individual’s age is between 25 and 65. The data cover the period between 1982 and 2021. $\rho$ is estimated by the sample analog of Eq. (3)

The left panel of Fig. 3 displays the correlation between the spouses’ employment over time. The reported correlation is a five year centered moving average. The correlation is always positive for all of the ethnicities. For Blacks and Whites, it remained more or less stable over time, while it decreased dramatically for the other groups, especially for Hispanics and for Others. It is difficult to compare correlations of different pairs of binary variables when the marginal probabilities differ across the pairs. In the right panel of Fig. 3, we therefore present the five year centered moving average of the estimate of the parameter $\rho$ in a Schmidt–Strauss model with no explanatory variables. Here ${{\hat{\rho }}}$ is calculated by the sample analog of Eq. (3). The estimated trend for $\rho$ is similar to that for the correlation, although $\rho$ shows a larger difference between Whites and Blacks.

### Static cross-sectional Schmidt–Strauss models

It is clear from the evidence in Sect. 4.1 that there is a strong relationship between employment of husbands and of wives. In this section, we document that this persists after controlling for a set of observable characteristics. Specifically, in the first four columns of Table 3, we present the results from estimating separate single-equation logit models for employment for husbands and for wives as well as the results from maximum likelihood estimation of the Schmidt–Strauss model in Eq. (2 ). The explanatory variables are dummy variables for the presence of children younger than 5, for any children, for the person’s own ethnicity, for the education categories “some college” and “college and above,” and dummy variables for the ethnicity of the couple. The estimation also controls for year dummies, the age and the age-squared of both the husband and the wife, as well as the interaction of the ages. The last four columns present the results from estimating the same models after also including the ethnicity and the education variables of the spouse as explanatory variables.

**Table 3:** Estimates of static cross-sectional models of employment

	Univariate logits		Schmidt–Strauss		Univariate logits		Schmidt–Strauss	
	Women	Men	Women	Men	Women	Men	Women	Men
Kids < 5	$$-0.808$$***	0.015*	$$-0.814$$***	0.146***	$$-0.801$$***	0.009	$$-0.804$$***	$$0.143^{***}$$
	(0.006)	(0.008)	(0.006)	(0.009)	(0.006)	(0.009)	(0.006)	(0.009)
Kids	$$-0.183$$***	0.218***	$$-0.209$$***	0.253 ***	$$-0.180$$***	0.220***	$$-0.206$$***	$$ 0.254^{***}$$
	(0.005)	(0.006)	(0.005)	(0.006)	(0.005)	(0.006)	(0.005)	(0.006)
Black (Woman)	$$-0.041$$		$$-0.055$$		$$-0.055$$	$$ -0.114$$*	$$-0.045$$	$$-0.107^{*}$$
	(0.045)		(0.045)		(0.052)	(0.063)	(0.053)	(0.064)
Hispanic (Woman)	$-0.095$***		$-0.111$***		$-0.132$ ***	$-0.003$	$-0.134$***	0.019
	(0.020)		(0.020)		(0.039)	(0.047)	(0.039)	(0.048)
Other (Woman)	$-0.156$***		$-0.171$***		$-0.199$ ***	$-0.078$*	$-0.195$***	$-0.047$
	(0.020)		(0.020)		(0.038)	(0.046)	(0.039)	(0.047)
Some college (Woman)	0.381***		0.365***		0.395 ***	0.188***	0.381***	$$0.124^{***}$$
	(0.005)		(0.005)		(0.005)	(0.007)	(0.005)	(0.007)
College+ (Woman)	0.571***		0.537***		0.699 ***	0.228***	0.687***	$$0.116^{***}$$
	(0.005)		(0.005)		(0.006)	(0.008)	(0.006)	(0.008)
Black (Man)		$$-0.376$$***		$$-0.392$$***	0.109 ***	$$-0.409$$***	0.153***	$$-0.433^{***}$$
		(0.033)		(0.033)	(0.039)	(0.046)	(0.040)	(0.047)
Hispanic (Man)		0.003		$$-0.018$$	$$-0.062$$	$$ -0.039$$	$$-0.060$$	$$-0.030$$
		(0.025)		(0.026)	(0.039)	(0.048)	(0.040)	(0.049)
Other (Man)		$$-0.192$$***		$$-0.214$$***	$$-0.109$$ ***	$$-0.239$$***	$$-0.086$$**	$$-0.225^{***}$$
		(0.028)		(0.028)	(0.040)	(0.048)	(0.041)	(0.049)
Some college (Man)		0.318***		0.298***	0.104 ***	0.259***	0.079***	$$0.247^{***}$$
		(0.006)		(0.006)	(0.005)	(0.007)	(0.005)	(0.007)
College+ (Man)		0.742***		0.699***	$$-0.219$$ ***	0.631***	$$-0.281$$***	$$0.676^{***}$$
		(0.006)		(0.006)	(0.006)	(0.007)	(0.006)	(0.007)
Black household	0.141***	$-0.134$***	0.220***	$-0.144$***	0.028	0.013	0.033	0.007
	(0.045)	(0.035)	(0.046)	(0.035)	(0.077)	(0.093)	(0.078)	(0.095)
Hispanic household	$$-0.419$$***	$$-0.147$$***	$$-0.392$$ ***	$$-0.028$$	$$-0.331$$***	$$-0.065$$	$$ -0.332 $$***	$$-0.006 $$
	(0.021)	(0.027)	(0.022)	(0.028)	(0.075)	(0.091)	(0.076)	(0.093)
Other household	$$-0.092$$***	$$-0.147$$***	$$-0.051$$**	$$-0.086$$***	0.079	$$-0.016$$	0.080	$$ -0.027$$
	(0.022)	(0.030)	(0.022)	(0.031)	(0.074)	(0.090)	(0.076)	(0.091)
Mixed household	0.002	$$-0.153$$***	0.029**	$$ -0.134$$***	0.042	$$-0.105$$**	0.052	$$ -0.113^{**} $$
	(0.012)	(0.015)	(0.012)	(0.015)	(0.038)	(0.046)	(0.039)	(0.047)
$\rho$				0.718***				0.730***
				(0.005)				(0.005)

***$$p<$$0.01, **$$p<$$0.05, *$$p<$$0.1

The dependent variable is working and the parameters are estimated by maximum likelihood. The data are from IPUMS CPS and cover a balanced panel of couples where each individual’s age is between 25 and 65. The data cover the period between 1982 and 2021. Coefficients on year dummies, husband’s and wife’s age, their interaction and their squares are not reported. Standard errors are clustered at the household level

The estimates of $\rho$ in Table 3 clearly suggest that there is positive association between the employment of husbands and wives after controlling for observed characteristics. In order to investigate whether this association varies systematically across ethnicities, we re-estimate the model in the last two columns of Table 3 separately for each ethnicity. In Table 4, we report the estimated $\rho$ ’s. The most striking finding is that the estimated $\rho$ for Whites is much larger than for other ethnicities, while the estimate for Hispanics is the lowest. This is also reflected in counterfactual marginal effects. Specifically, for each ethnicity, we calculate the average probabilities implied by the model that a wife works conditional on whether her husband works or not. The difference in these average probabilities is 18 percentage points for Whites, 8 for Hispanics, and between 11 and 14 for each of the other three groups. The corresponding counterfactual marginal effects for husbands are 10 percentage points for Whites, 4 for Hispanics, and between 6 and 9 percentage points for the other groups. This ordering is consistent with that found in Fig. 3.

**Table 4:** Estimates of $\rho$ in the static cross-sectional Schmidt–Strauss model by household ethnicity

	White	Black	Hispanic	Other	Mixed
$$\rho $$	0.814***	0.540***	0.356***	0.604 ***	$$0.506^{***}$$
	(0.006)	(0.019)	(0.019)	(0.025)	(0.020)

***$$p<$$0.01, **$$p<$$0.05, *$$p<$$0.1

The dependent variable is working and the parameters are estimated by maximum likelihood using the same specification as in Table 3. The data are from IPUMS CPS and cover a balanced panel of couples where each individual’s age is between 25 and 65. The data cover the period between 1982 and 2021. Standard errors are clustered at the household level

**Fig. 4:**
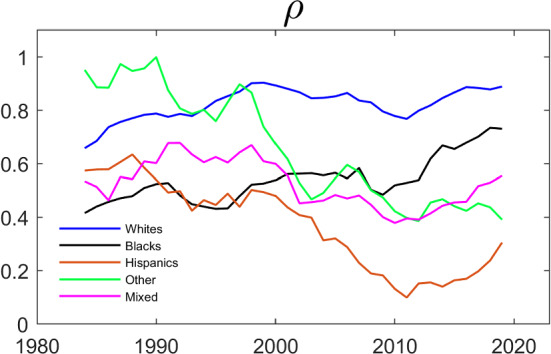
Evolution of $\rho$ over Time by Household Ethnicity. The dependent variable is working and the parameters are estimated by maximum likelihood using the same specification as in Table 3. The data are from IPUMS CPS and cover a balanced panel of couples where each individual’s age is between 25 and 65. The data cover the period between 1982 and 2021 and the estimation is done over five year centered rolling windows

Figure 3 above suggested a dramatic fall in the association between the employment of wives and husbands for households where both the wife and the husband are Hispanic, and for households where each spouse is of “other ethnicity”. To investigate whether this holds after controlling for observable covariates, we estimate the model in the last two columns of Table 3 for each ethnicity and for rolling 5-year time-spans. The estimated $\rho$ coefficients are presented in Fig. 4. Qualitatively, the pattern in Fig. 4 is similar to that in Fig. 3: The association between the employment of wives and husbands has been falling for Hispanics and for Others, while it has been relatively stable for White, Black and Mixed couples.

### Dynamic panel data Schmidt–Strauss models

In the Schmidt–Strauss models estimated in Table 3, the only avenue for interdependence between the employment of wives and husbands (conditional on the observed characteristics) is through the parameter $\rho$. If the employment of a partner actually also depends on the lagged employment of both partners, then this will be captured by the estimate of $\rho$.

In order to investigate the role of dynamics, we first estimate the Schmidt–Strauss model in the last two columns of Table 3 after including an individual’s own as well as the partner’s lagged employment as explanatory variables. Specifically, we estimate the model

4 $$\begin{aligned}{} & {} P\left( \left. y_{1,it}=c_{1},y_{2,it}=c_{2}\right| \left\{ y_{1,is},y_{2,is}\right\} _{s<t},\left\{ x_{1,is}\right\} _{s=1}^{t},\left\{ x_{2,is}\right\} _{s=1}^{t}\right) \\= & {} \frac{\exp \left( c_{1}\left( z_{1,it}\right) +c_{2}\left( z_{2,it}\right) +c_{1}c_{2}\rho \right) }{1+\exp \left( z_{1,it}\right) +\exp (z_{2,it})+\exp \left( z_{1,it}+z_{2,it}+\rho \right) } \nonumber \end{aligned}$$

for $$c_{1},c_{2}\in \left\{ 0,1\right\} $$, where

$$\begin{aligned} z_{1,it}= & {} x_{1,it}^{\prime }\beta _{1}+y_{1,it-1}\gamma _{11}+y_{2,it-1}\gamma _{12} \\ z_{2,it}= & {} x_{2,it}^{\prime }\beta _{2}+y_{1,it-1}\gamma _{21}+y_{2,it-1}\gamma _{22}. \end{aligned}$$

The results are presented in Table 5. Since the lagged values of the dependent variable are not observed in the first time period, we do the estimation using waves two through four of our dataset. The results in Table 5 suggest that each partner’s employment depends strongly and positively on her or his own lagged employment, and that it depends negatively on the partner’s lagged employment (after controlling for the observed covariates). In combination, these will introduce a negative correlation in the contemporaneous employment status, which - in turn - would lead to a downward bias in the estimate of $\rho$ when these dynamic interactions are not controlled for in the model. This is reflected in the higher estimate of $\rho$ in the model that allows for lagged employment of both partners as explanatory variables as in Eq. (4).

**Table 5:** Estimates of dynamic Schmidt–Strauss models of employment

	Schmidt–Strauss	
	Women	Men
Lagged employment (Woman)	4.684***	$$-1.668^{***}$$
	(0.005)	(0.008)
Lagged employment (Man)	$$-1.668$$***	$$4.343^{***}$$
	(0.008)	(0.006)
Kids < 5	$$-0.425$$***	$$0.067^{***}$$
	(0.006)	(0.007)
Kids	$$-0.110$$***	$$0.162^{***}$$
	(0.004)	(0.005)
Black (Woman)	$-0.049$	$-0.028$
	(0.047)	(0.052)
Hispanic (Woman)	$-0.097$***	0.037
	(0.035)	(0.040)
Other (Woman)	$-0.115$***	$-0.004$
	(0.034)	(0.038)
Some college (Woman)	0.213***	$$0.077^{***}$$
	(0.005)	(0.006)
College+ (Woman)	0.382***	$$0.077^{***}$$
	(0.006)	(0.006)
Black (Man)	0.055	$$-0.223^{***}$$
	(0.035)	(0.039)
Hispanic (Man)	$-0.041$	0.011
	(0.035)	(0.040)
Other (Man)	$$-0.054$$	$$-0.111^{***}$$
	(0.036)	(0.041)
Some college (Man)	0.036***	$$0.151^{***}$$
	(0.005)	(0.006)
College+ (Man)	$$-0.159$$***	$$0.393^{***}$$
	(0.005)	(0.006)
Black household	0.076	$-0.077$
	(0.069)	(0.077)
Hispanic household	$-0.160$**	$-0.068$
	(0.067)	(0.076)
Other household	0.079	$-0.066$
	(0.066)	(0.075)
Mixed household	0.059*	$$-0.102^{***}$$
	(0.034)	(0.039)
$$\rho $$	$$2.040^{***}$$	
	(0.008)	

***$$p<$$0.01, **$$p<$$0.05, *$$p<$$0.1

The dependent variable is working and the parameters are estimated by maximum likelihood. The data are from IPUMS CPS and cover a balanced panel of couples where each individual’s age is between 25 and 65. The data cover the period between 1982 and 2021. Coefficients on year dummies, husband’s and wife’s age, their interaction and their squares are not reported. Standard errors are clustered at the household level

Since controlling for the lagged employment status of both partners dramatically change the estimate of $\rho$ when we use the full sample, we next investigate whether the same is true across ethnicities. Specifically, we estimate the same specification as in Table 5 separately for each ethnicity group. Table 6 reports the estimated coefficients on the lagged employment variables as well as the estimated $\rho$. In this specification, Hispanics and Blacks are quite similar to each other in terms of the contemporaneous interdependence between the employment status of the two partners (measured by $\rho$) as well as in terms of the dynamic interdependence (measured by the $\gamma$’s).

**Table 6:** Estimates of dynamic Schmidt–Strauss models of employment by household ethnicity

	All	Whites	Blacks	Hispanics	Other	Mixed
$$\gamma _{11}$$	4.684***	4.678***	4.481***	4.716***	4.976***	$$4.678^{***}$$
	(0.005)	(0.006)	(0.023)	(0.021)	(0.029)	(0.022)
$$\gamma _{12}$$	$$-1.668$$***	$$-1.759$$***	$$-1.041$$***	$$-1.096$$***	$$-1.475$$***	$$-1.629^{***}$$
	(0.008)	(0.009)	(0.037)	(0.037)	(0.050)	(0.034)
$$\gamma _{21}$$	$$-1.668$$***	$$-1.759$$***	$$-1.061$$***	$$-1.082$$***	$$-1.460$$***	$$-1.638^{***}$$
	(0.008)	(0.009)	(0.037)	(0.036)	(0.050)	(0.034)
$$\gamma _{22}$$	4.343***	4.363***	4.344***	4.019***	4.470***	$$4.359^{***}$$
	(0.006)	(0.007)	(0.024)	(0.023)	(0.032)	(0.025)
$$\rho $$	2.040***	2.170***	1.357***	1.262 ***	1.764***	$$1.868^{***}$$
	(0.008)	(0.009)	(0.037)	(0.037)	(0.050)	(0.033)

***$$p<$$0.01, **$$p<$$0.05, *$$p<$$0.1

The dependent variable is working and the parameters are estimated by maximum likelihood using the same specification as in Table 5. The data are from IPUMS CPS and cover a balanced panel of couples where each individual’s age is between 25 and 65. The data cover the period between 1982 and 2021. Standard errors are clustered at the household level

**Fig. 5:**
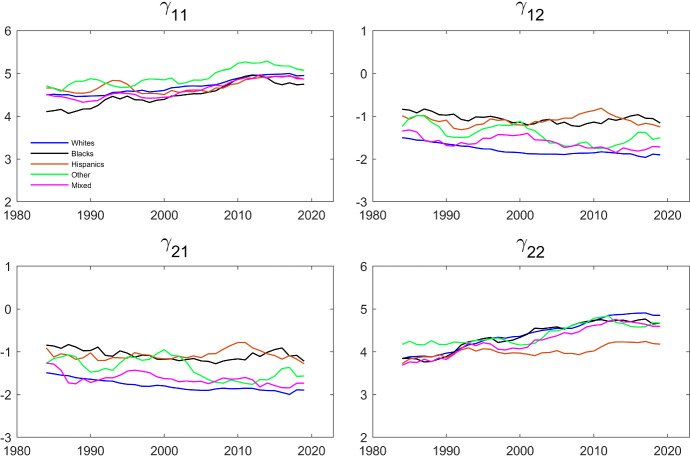
Evolution of $\gamma$’s over Time by Household Ethnicity. The dependent variable is working and the parameters are estimated by maximum likelihood using the same specification as in Table 5. The data are from IPUMS CPS and cover a balanced panel of couples where each individual’s age is between 25 and 65. The data cover the period between 1982 and 2021 and the estimation is done over five year centered rolling windows

**Fig. 6:**
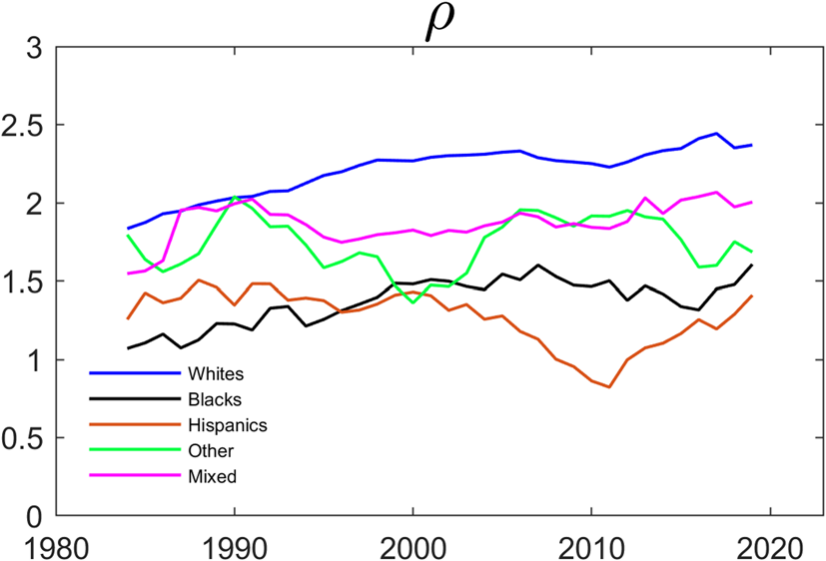
Evolution of $\rho$ over Time by Household Ethnicity. The dependent variable is working and the parameters are estimated by maximum likelihood using the same specification as in Table 5. The data are from IPUMS CPS and cover a balanced panel of couples where each individual’s age is between 25 and 65. The data cover the period between 1982 and 2021 and the estimation is done over five year centered rolling windows

The evolution of the estimates of the parameters that govern the dynamics and the interdependence is shown in Figs. 5 and 6. Specifically, we estimate the Schmidt–Strauss model in Table 5 for each ethnicity over rolling 5-year time-spans and plotted the estimates of the $\gamma$’s and of $\rho$ against time. Comparing the patterns in Fig. 6 to the patterns in Fig. 4 , we see that Black and Hispanic couples are more similar. This is consistent with the finding in Table 6. Interestingly, the estimated $\rho$’s for Hispanics and for Others are now much more stable over time, while the $\rho$ for Whites is now trending up.

It is well-understood that it can be difficult to disentangle state dependence (the causal dependence of a variable at one point in time from its value in the previous period) from unobserved heterogeneity. Intuition suggests that it is also difficult to distinguish between the effect of $\rho$ and the effect of unobserved heterogeneity that is correlated between the husband and wife in the same household. These issues raise the question of whether it is possible to semiparametrically identify $\rho$ and the coefficients on the lagged dependent variables in a model that allows for fixed effects. In the next section, we therefore investigate whether it is possible to identify and estimate the parameters of a model that allows for fixed effects in the dynamic Schmidt–Strauss framework.

## Dynamic panel data Schmidt–Strauss models with fixed effects

Honoré and Kyriazidou (2019) adapt the Schmidt–Strauss model discussed in Sect. 2 to a static panel data setting where each outcome can also depend on unit-specific fixed effects. Specifically, they assume that

5 $$\begin{aligned}&P\left( \left. y_{1,it}=1\right| y_{2,it},\left\{ y_{1,is},y_{2,is}\right\} _{s<t},\left\{ x_{1,is}\right\} _{s=1}^{T},\left\{ x_{2,is}\right\} _{s=1}^{T},\alpha _{1,i},\alpha _{2,i}\right) \nonumber \\&=\varLambda \left( \alpha _{1,i}+x_{1,it}^{\prime }\beta _{1}+\rho y_{2,it}\right) \end{aligned}$$

and

6 $$\begin{aligned}&P\left( \left. y_{2,it}=1\right| y_{1,it},\left\{ y_{1,is},y_{2,is}\right\} _{s<t},\left\{ x_{1,is}\right\} _{s=1}^{T},\left\{ x_{2,is}\right\} _{s=1}^{T},\alpha _{1,i},\alpha _{2,i}\right) \\&=\varLambda \left( \alpha _{2,i}+x_{2,it}^{\prime }\beta _{2}+\rho y_{1,it}\right) \nonumber \end{aligned}$$

In this model, $$\alpha _{1,i}$$ and $$\alpha _{2,i}$$ are the fixed effects, $$ x_{1,it}$$ and $$x_{2,it}$$ are strictly exogenous explanatory variables, and $$ \rho $$ is the cross-equation dependence parameter, which as in Schmidt and Strauss (1975), needs to be the same in the two equations given the structure in equations (5) and (6).

Following Schmidt and Strauss (1975), it can be shown that

$$\begin{aligned}&P\left( \left. y_{1,it}=c_{1},y_{2,it}=c_{2}\right| \left\{ y_{1,is},y_{2,is}\right\} _{s<t},\left\{ x_{1,is}\right\} _{s=1}^{T},\left\{ x_{2,is}\right\} _{s=1}^{T},\alpha _{1,i},\alpha _{2,i}\right) \\&=\frac{\exp \left( c_{1}\left( \alpha _{1,i}+x_{1,it}^{\prime }\beta _{1}\right) +c_{2}\left( \alpha _{2,i}+x_{2,it}^{\prime }\beta _{2}\right) +c_{1}c_{2}\rho \right) }{1+\exp \left( \alpha _{1,i}+x_{1,it}^{\prime }\beta _{1}\right) +\exp (\alpha _{2,i}+x_{2,it}^{\prime }\beta _{2})+\exp \left( \alpha _{1,i}+x_{1,it}^{\prime }\beta _{1}+\alpha _{2,i}+x_{2,it}^{\prime }\beta _{2}+\rho \right) } \end{aligned}$$

for $c_{1},c_{2}\in \left\{ 0,1\right\} .$ Honoré and Kyriazidou (2019) show that a conditional likelihood argument can be used to identify and estimate $\beta _{1}$, $\beta _{2}$, and $\rho$ with as few as $T=2$ time periods. Indeed, $\rho$ can be allowed to be time dependent in Eqs. (5) and (6).

Honoré and Kyriazidou (2019) also consider a vector autoregressive simultaneous logit model:

7 $$\begin{aligned} P\left( \left. y_{1,it}=1\right| y_{2,it},y_{1,i}^{t-1},y_{2,i}^{t-1},\alpha _{1i},\alpha _{2i}\right)= & {} \varLambda \left( \alpha _{1i}+y_{1,it-1}\gamma _{11}+y_{2,it-1}\gamma _{12}+\rho y_{2,it}\right) , \\ P\left( \left. y_{2,it}=1\right| y_{1,it},y_{1,i}^{t-1},y_{2,i}^{t-1},\alpha _{1i},\alpha _{2i}\right)= & {} \varLambda \left( \alpha _{2i}+y_{1,it-1}\gamma _{21}+y_{2,it-1}\gamma _{22}+\rho y_{1,it}\right) . \nonumber \end{aligned}$$

This model is arguably the most relevant fixed effects specification for the application in this paper. For each individual, we only use data from four months, so with the exception of time-dummies, there is essentially no exogenous variability in the explanatory variables over time. Moreover, we use one time period to provide the initial conditions, and the effect of time variables is probably not important over a three month period.^3^

Honoré and Kyriazidou (2019) show that $\left( \gamma _{11},\gamma _{12},\gamma _{21},\gamma _{22}\right)$ is identified in the model given in Eq. (7) with a total of four time periods (including the one that delivers the initial condition). However, the conditioning argument that leads to the identification eliminates the parameter $\rho$ along with the fixed effects, $\alpha _{1i}$ and $\alpha _{2i}$. On the positive side, this implies that one can allow the parameter $\rho$ in Eq. (7) to be individual-specific. On the other hand, $\rho$ may be the parameter of interest in many applications, including the one considered here. This makes it problematic that the conditioning argument eliminates it along with $\alpha _{1i}$ and $\alpha _{2i}$. In the next subsection, we first generalize the results in Honoré and Kyriazidou (2019) to show that using a conditional likelihood approach to eliminate $\alpha _{1i}$ and $\alpha _{2i}$ in Eq. (7) will also eliminate $\rho$ for all values of *T*. The conditional likelihood approach is then illustrated empirically by obtaining estimates of the $\gamma$’s in Eq. (7) in the context of husbands’ and wives’ employment. Since the simultaneity parameter, $\rho$, is not generally identified from a conditional likelihood approach, we next consider a restricted version of the model, in which the two individual fixed effects are the same, except for an additive constant which is the same across all pairs. In our application, we interpret this as a model with household specific fixed effects. This model is also illustrated empirically.

### Conditional likelihood for dynamic Schmidt–Strauss model with fixed effects

The traditional approach to estimating nonlinear fixed effects models is to find a sufficient statistic for the fixed effects, and then to construct a conditional likelihood function conditioning on the sufficient statistic. By construction, this conditional likelihood function will not depend on the fixed effects and it may or may not depend on some or all of the parameters of interest. In this subsection, we consider the conditional likelihood approach for the model in Eq. (7). This extends the analysis in Honoré and Kyriazidou (2019).

We consider a situation in which a pair of outcomes^4^$\left( y_{1,t},y_{2,t}\right)$ from Eq. (7) are observed for *T* periods. We also assume that the initial condition, $\left( y_{1,0},y_{2,0}\right)$, is observed. We denote the probability distribution of $\left( y_{1,0},y_{2,0}\right)$ by $p\left( y_{1,0},y_{2,0},\alpha _{1},\alpha _{2}\right)$, and we do not assume that it is necessarily generated by the same model. For notational simplicity, we let $z_{1,t}=\gamma _{11}y_{1,t}+\gamma _{12}y_{2,t}$ and $z_{2,t}=\gamma _{21}y_{1,t}+\gamma _{22}y_{2,t}$.

With this, the probability of a particular sequence is

$$\begin{aligned}{} & {} \frac{p\left( y_{1,0},y_{2,0},\alpha _{1},\alpha _{2}\right) \prod \limits _{t=1}^{T}\exp \left( y_{1,t}\left( z_{1,t-1}+\alpha _{1}\right) \right) \exp \left( y_{2,t}\left( z_{2,t}+\alpha _{2}\right) \right) \exp \left( y_{1,t}y_{2,t}\rho \right) }{\prod \limits _{t=1}^{T}\left\{ 1+\exp \left( z_{1,t-1}+\alpha _{1}\right) +\exp \left( z_{2,t-1}+\alpha _{2}\right) +\exp \left( z_{1,t-1}+z_{2,t-1}+\alpha _{1}+\alpha _{2}+\rho \right) \right\} } \\{} & {} \\= & {} \frac{p\left( y_{1,0},y_{2,0},\alpha _{1},\alpha _{2}\right) }{1+\exp \left( z_{1,0}+\alpha _{1}\right) +\exp \left( z_{2,0}+\alpha _{2}\right) +\exp \left( z_{1,0}+z_{2,0}+\alpha _{1}+\alpha _{2}+\rho \right) } \\{} & {} \frac{\prod \limits _{t=1}^{T}\exp \left( y_{1,t}\left( z_{1,t-1}+\alpha _{1}\right) \right) \exp \left( y_{2,t}\left( z_{2,t}+\alpha _{2}\right) \right) \exp \left( y_{1,t}y_{2,t}\rho \right) }{\prod \limits _{t=1}^{T-1} \left\{ 1+\exp \left( z_{1,t}+\alpha _{1}\right) +\exp \left( z_{2,t}+\alpha _{2}\right) +\exp \left( z_{1,t}+z_{2,t}+\alpha _{1}+\alpha _{2}+\rho \right) \right\} }. \end{aligned}$$

Now consider two different sequences of $\left\{ \left( y_{1,t},y_{2,t}\right) \right\} _{t=1}^{T}$ with the same $\left( y_{1,0},y_{2,0}\right)$. The probability of one of the sequences conditional on observing one of the two depends on the ratio of the probabilities for the two sequences. The key question is whether the individual-specific effects cancel in that ratio.

In the numerator, the $\alpha$’s cancel if two sequences have the same $\sum _{t=1}^{T}y_{1,t}$ and the same $\sum _{t=1}^{T}y_{2,t}$. In the denominator, each combination of $\left( y_{1,t}y_{2,t}\right)$ must appear equally often. The latter is the same as saying that $\sum _{t=1}^{T-1}y_{1,t}$, $\sum _{t=1}^{T-1}y_{2,t}$, $\sum _{t=1}^{T-1}y_{1,t}y_{2,t}$ must be the same^5^. This suggests the sufficient statistic

$$\begin{aligned} \left( y_{1,0},y_{2,0},\sum _{t=1}^{T-1}y_{1,t}\text {, } \sum _{t=1}^{T-1}y_{2,t},\sum _{t=1}^{T-1}y_{1,t}y_{2,t},y_{1,T},y_{2,T}\right) \end{aligned}$$

and the conditional likelihood function (for a given observation with fixed effects $\alpha _{1}$ and $\alpha _{2}$) is therefore

8 $$\begin{aligned} \mathcal {L=}\frac{\prod \limits _{t=1}^{T}\exp \left( y_{1,t}\left( \gamma _{11}y_{1,t-1}+\gamma _{12}y_{2,t-1}\right) \right) \exp \left( y_{2,t}\left( \gamma _{21}y_{1,t-1}+\gamma _{22}y_{2,t-1}\right) \right) \ }{ \sum \limits _{{\mathcal {B}}}\prod \limits _{t=1}^{T}\exp \left( c_{t}\left( \gamma _{11}c_{t-1}+\gamma _{12}d_{t-1}\right) \right) \exp \left( d_{t}\left( \gamma _{21}c_{t-1}+\gamma _{22}d_{t-1}\right) \right) \ }, \end{aligned}$$

where ${\mathcal {B}}$ is the set of all sequences, $\{c_t,d_t\}_{t=0}^T$, such that

$$\begin{aligned} (c_{0},d_{0})\!=\!(y_{1\!,0},y_{2\!,0}),\sum _{t=1}^{T-1}c_{t}\!=\! \sum _{t=1}^{T-1}y_{1\!,t},\sum _{t=1}^{T-1}d_{t}\!=\!\sum _{t=1}^{T-1}y_{2\!,t}, \\ \sum _{t=1}^{T-1}c_{t}d_{t}\!=\! \sum _{t=1}^{T-1}y_{1\!,t}y_{2\!,t},(c_{T},d_{T})\!=\!(y_{1\!,T},y_{2\!,T}). \end{aligned}$$

Note that not only does $\alpha$ drop out of the conditional likelihood, but so does $\rho$. In other words, a conditional likelihood approach does not identify $\rho$ for any *T*. Also note that the conditional likelihood is constant if $T<3$, so at least three periods are needed in addition to the one providing the initial conditions.

We finally note that the argument above is unchanged if one replaces $\gamma _{11}$, $\gamma _{12}$, $\gamma _{21}$, $\gamma _{22}$, and $\rho$ with functions of exogenous covariates as long as the functions do not change over time. For example, in the application some of these parameters could be functions of the level of education or of the presence of children.

### Empirical illustration

**Fig. 7:**
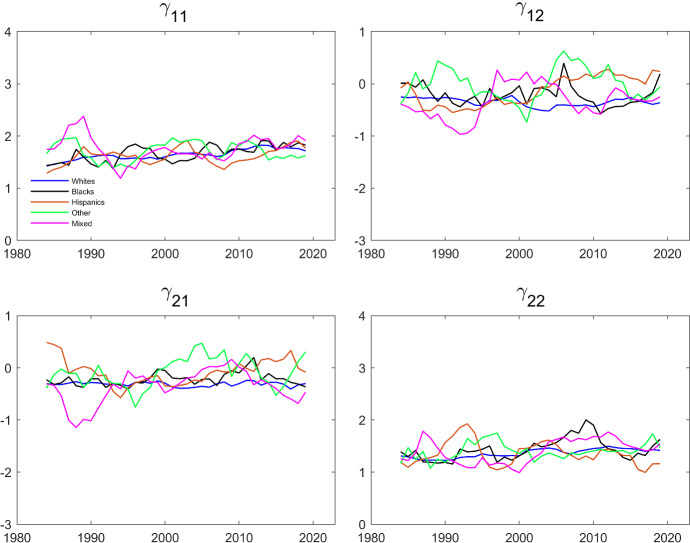
Evolution of $\gamma$’s over Time by Household Ethnicity (Fixed Effects). The dependent variable is working and the parameters are estimated maximizing the conditional likelihood in Eq. (8). The data are from IPUMS CPS and cover a balanced panel of couples where each individual’s age is between 25 and 65. The data cover the period between 1982 and 2021 and the estimation is done over five year centered rolling windows

In Table 7, we present the results from estimating $\gamma _{11}$, $\gamma _{12}$, $\gamma _{21}$, and $\gamma _{22}$ using the conditional likelihood approach discussed above for the full sample as well as by ethnicity. As one might expect, these parameters are much lower in the fixed effects specification than those reported in Table 6, where we do not allow for unobserved heterogeneity. Figure 7 shows the results of estimating the model on rolling 5-year sub-samples for each ethnicity. The estimates are fairly stable over time, and not very different across ethnicities. Overall, there is strong evidence that, after controlling for fixed effects, an individual’s own lagged employment has a positive effect. The effect of the spouse’s lagged employment tends to be negative and smaller in magnitude. As a comparison, Chountas and Kyriazidou (2021) estimate multinomial fixed effects model of husbands and wives employment. They use quarterly data from the German Socio-Economic panel for the years 2013–15 and four different labor states (full time employment, part time employment, unemployment and out of labor force), and find strong negative effects of the husband’s lagged employment on the wife, but mostly positive although statistically insignificant effects of the wife’s lagged employment on the husband.

**Table 7:** Estimates of dynamic Schmidt–Strauss model with fixed effects by household ethnicity

	All	White	Black	Hispanic	Other	Mixed
$$\gamma _{11}$$	1.620***	1.611***	$$1.640^{***}$$	1.601***	1.684***	$$1.761^{***}$$
	(0.014)	(0.016)	(0.061)	(0.056)	(0.080)	(0.062)
$$\gamma _{12}$$	$$-0.296 $$***	$$-0.338 $$***	$$-0.193 $$ **	$$-0.078 $$	$$-0.030 $$	$$-0.268^{***}$$
	(0.022)	(0.025)	(0.085)	(0.085)	(0.113)	(0.088)
$$\gamma _{21}$$	$$-0.280 $$***	$$-0.311 $$***	$$-0.246 $$ ***	$$-0.039 $$	$$-0.079 $$	$$-0.302^{***}$$
	(0.021)	(0.024)	(0.086)	(0.080)	(0.112)	(0.085)
$$\gamma _{22}$$	1.357***	1.350***	1.415***	1.324***	1.381***	$$1.420^{***}$$
	(0.017)	(0.019)	(0.065)	(0.058)	(0.086)	(0.067)

***$$p<$$0.01, **$$p<$$0.05, *$$p<$$0.1

The dependent variable is working and the parameters are estimated maximizing the conditional likelihood in Eq. (8). The data are from IPUMS CPS and cover a balanced panel of couples where each individual’s age is between 25 and 65. The data cover the period between 1982 and 2021

### Conditional likelihood for dynamic Schmidt–Strauss model with restricted fixed effects

In this subsection, we investigate whether additional identification can be obtained by assuming that $\alpha _{1}=\alpha$ and $\alpha _{2}=\alpha +\kappa$ for some constant $\kappa$, which does not vary across units. Our motivation is to see whether this will allow for identification of $\rho$. In our application, we interpret this as a model with a family specific fixed effect ($\alpha$) and a spouse specific level ($\kappa$).

As before, we consider a situation in which a pair of outcomes from Eq. (7) are observed for *T* periods (in addition to period 0, which delivers the initial condition). Again, we use the notation $z_{1,t}=\gamma _{11}y_{1,t}+\gamma _{12}y_{2,t}$ and $z_{2,t}=\gamma _{21}y_{1,t}+\gamma _{22}y_{2,t}$. With $\alpha _{2}=\alpha +\kappa$ , the probability of a particular sequence becomes

$$\begin{aligned}{} & {} \frac{p\left( y_{1,0},y_{2,0},\alpha \right) \prod \limits _{t=1}^{T}\exp \left( y_{1,t}\left( z_{1,t-1}+\alpha \right) \right) \exp \left( y_{2,t}\left( z_{2,t}+\alpha +\kappa \right) \right) \exp \left( y_{1,t}y_{2,t}\rho \right) }{\prod \limits _{t=1}^{T}\left\{ 1+\exp \left( z_{1,t-1}+\alpha \right) +\exp \left( z_{2,t-1}+\alpha +\kappa \right) +\exp \left( z_{1,t-1}+z_{2,t-1}+2\alpha +\kappa +\rho \right) \right\} } \\{} & {} \\= & {} \frac{p\left( y_{1,0},y_{2,0},\alpha \right) }{1+\exp \left( z_{1,0}+\alpha \right) +\exp \left( z_{2,0}+\alpha +\kappa \right) +\exp \left( z_{1,0}+z_{2,0}+2\alpha +\kappa +\rho \right) } \\{} & {} \frac{\prod \limits _{t=1}^{T}\exp \left( y_{1,t}\left( z_{1,t-1}+\alpha \right) \right) \exp \left( y_{2,t}\left( z_{2,t}+\alpha +\kappa \right) \right) \exp \left( y_{1,t}y_{2,t}\rho \right) }{\prod \limits _{t=1}^{T-1} \left\{ 1+\exp \left( z_{1,t}+\alpha \right) +\exp \left( z_{2,t}+\alpha +\kappa \right) +\exp \left( z_{1,t}+z_{2,t}+2\alpha +\kappa +\rho \right) \right\} }. \end{aligned}$$

As above, the key question is whether the unit-specific *a*’s cancel in the ratio of the probabilities of two different sequences with the same initial conditions. In the numerator, the $\alpha$’s cancel if the two sequences have the same $\sum _{t=1}^{T}y_{1,t}+\sum _{t=1}^{T}y_{2,t}$. In the denominator, each combination of $\left( y_{1,t},y_{2,t}\right)$ must appear equally often^6^. The latter is the same as saying that $\sum _{t=1}^{T-1}y_{1,t}$, $\sum _{t=1}^{T-1}y_{2,t}$, $\sum _{t=1}^{T-1}y_{1,t}y_{2,t}$ must be the same. This suggests the sufficient statistic

$$\begin{aligned} \left( y_{1,0},y_{2,0},\sum _{t=1}^{T-1}y_{1,t},\sum _{t=1}^{T-1}y_{2,t}, \sum _{t=1}^{T-1}y_{1,t}y_{2,t},y_{1,T}+y_{2,T}\right) \end{aligned}$$

The difference from the case where the $\alpha$’s are unrestricted is that we do not need to condition on $y_{1,T}$ and $y_{2,T}$, but only on the sum. The implication is that a conditional likelihood approach will lead to more sequences being compared to each other.

The conditional likelihood function (for a given individual) is

9 $$\begin{aligned} {\mathcal {L}}=\frac{\prod \limits _{t=1}^{T}\exp \left( y_{1,t}\left( \gamma _{11}y_{1,t-1}+\gamma _{12}y_{2,t-1}\right) \right) \exp \left( y_{2,t}\left( \gamma _{21}y_{1,t-1}+\gamma _{22}y_{2,t-1}+\kappa \right) \right) }{\sum \limits _{{\mathcal {B}}}\prod \limits _{t=1}^{T}\exp \left( c_{t}\left( \gamma _{11}c_{t-1}+\gamma _{12}d_{t-1}\right) \right) \exp \left( d_{t}\left( \gamma _{21}c_{t-1}+\gamma _{22}d_{t-1}+\kappa \right) \right) } \nonumber \\ \end{aligned}$$

where ${\mathcal {B}}$ is the set of all sequences, $\{c_{t},d_{t}\}_{t=0}^{T}$ , such that

$$\begin{aligned}{} & {} (c_{0},d_{0})\!=\!(y_{1\!,0},y_{2\!,0}),\sum _{t=1}^{T-1}c_{t}\!=\! \sum _{t=1}^{T-1}y_{1\!,t},\sum _{t=1}^{T-1}d_{t}\!=\!\sum _{t=1}^{T-1}y_{2\!,t},\\{} & {} \sum _{t=1}^{T-1}c_{t}d_{t}\!=\! \sum _{t=1}^{T-1}y_{1\!,t}y_{2\!,t},c_{T}+d_{T}\!=\!y_{1\!,T}+y_{2\!,T}. \end{aligned}$$

Note that while $\alpha$ and $\rho$ drop out of this expression, $\kappa$ does not. Also note that this argument is unchanged if one replaces $\kappa$ with some function of predetermined covariates as long as the function does not change over time. The same is true for the parameters $\gamma _{11}$, $\gamma _{12}$, $\gamma _{21}$, and $\gamma _{22}$.

### Empirical illustration

In Table 8, we present the results from estimating $\gamma _{11}$, $\gamma _{12}$, $\gamma _{21}$, and $\gamma _{22}$ using the conditional likelihood approach discussed above for the full sample as well as by ethnicity. The fixed effects estimates are again lower than those reported in Table 6, which did not allow for unobserved heterogeneity, but they are larger than the ones that were obtained when we did not restrict the fixed effects for the husbands and the wives reported in Table 7. Since the conditional likelihood in Eq. (9) uses more observations that the one in Eq. (8), we would expect the estimated standard error to be smaller in Table 8 than in Table 7.

**Fig. 8:**
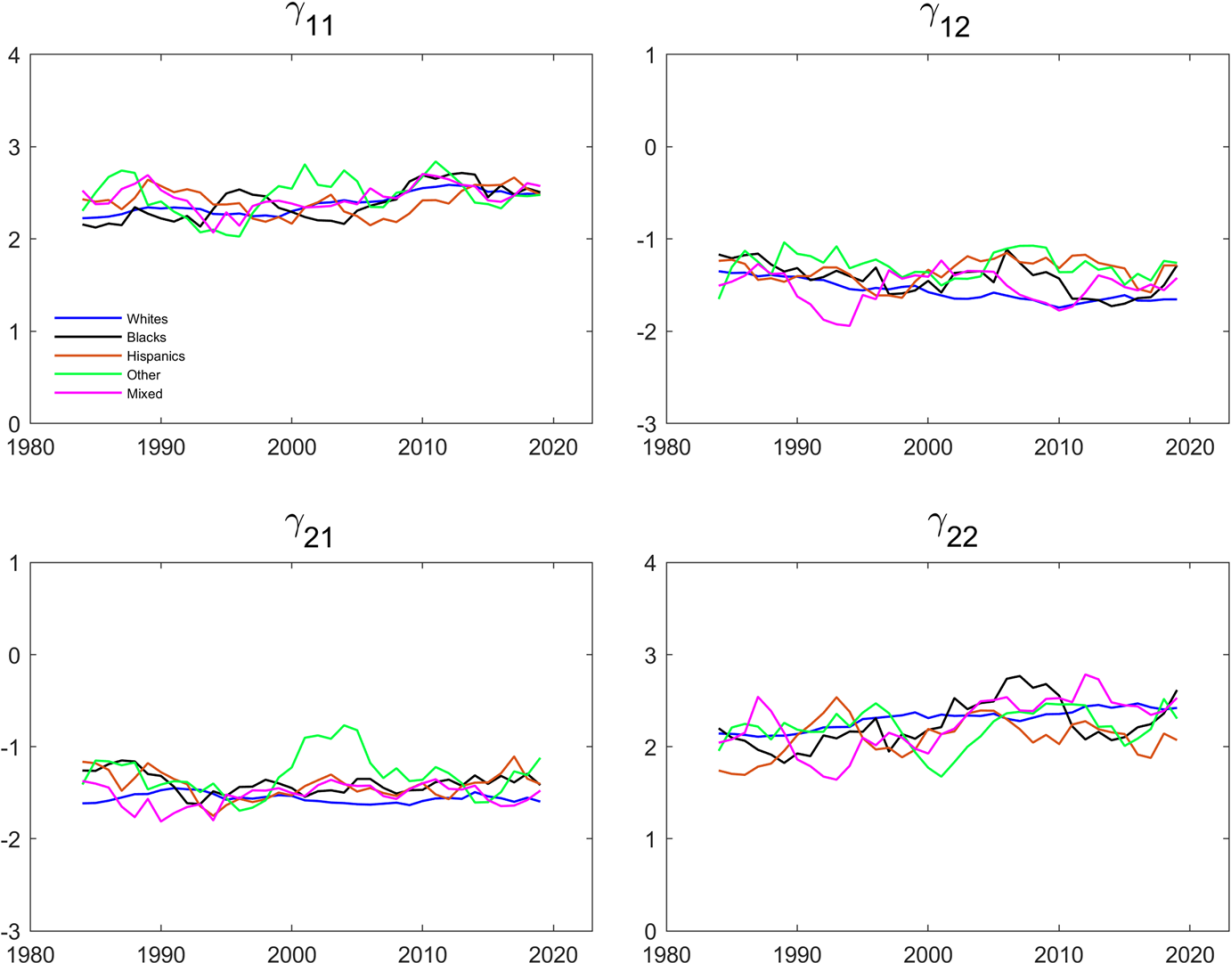
Evolution of $\gamma$’s over Time by Household Ethnicity (Restricted Fixed Effects). The dependent variable is working and the parameters are estimated maximizing the conditional likelihood in Eq. (9). The data are from IPUMS CPS and cover a balanced panel of couples where each individual’s age is between 25 and 65. The data cover the period between 1982 and 2021 and the estimation is done over five year centered rolling windows

Figure 8 shows the results of estimating the model on rolling 5-year sub-samples for each ethnicity. The estimates are fairly stable over time, and not very different across ethnicities.

**Table 8:** Estimates of dynamic Schmidt–Strauss model with restricted fixed effects by household ethnicity

	All	Whites	Blacks	Hispanics	Other	Mixed
$$\gamma _{11}$$	2.385***	2.374***	2.364***	2.403***	2.436***	$$2.485^{***}$$
	(0.014)	(0.016)	(0.058)	(0.059)	(0.078)	(0.060)
$$\gamma _{12}$$	$$-1.511 $$***	$$-1.542 $$***	$$-1.409 $$ ***	$$-1.318 $$***	$$-1.298 $$***	$$-1.505^{***}$$
	(0.015)	(0.018)	(0.063)	(0.060)	(0.081)	(0.065)
$$\gamma _{21}$$	$$-1.538 $$***	$$-1.576 $$***	$$-1.392 $$ ***	$$-1.403 $$***	$$-1.310 $$***	$$-1.485^{***}$$
	(0.015)	(0.016)	(0.060)	(0.060)	(0.078)	(0.061)
$$\gamma _{22}$$	2.263***	2.285***	2.231***	2.097***	2.206***	$$2.315^{***}$$
	(0.016)	(0.018)	(0.064)	(0.059)	(0.083)	(0.065)

***$$p<$$0.01, **$$p<$$0.05, *$$p<$$0.1

The dependent variable is working and the parameters are estimated maximizing the conditional likelihood in Eq. (9). The data are from IPUMS CPS and cover a balanced panel of couples where each individual’s age is between 25 and 65. The data cover the period between 1982 and 2021

## Moment conditions for the dynamic Schmidt–Strauss model with fixed effects

In panel data models with fixed effects, it is sometimes possible to construct moment conditions that do not depend on the fixed effects. When that is the case, one can consider estimating the common parameters of the model by generalized method of moments. The dynamic linear panel data model is a simple example of this; see, for example Anderson and Hsiao (1981) or Holtz Eakin et al. (1988). Applications of this idea to nonlinear models include Honoré (1992), Kyriazidou (2001), Hu (2002) and Kitazawa (2013).^7^ Bonhomme (2012) proposes a general approach for constructing such moment conditions and Honoré and Weidner (2022) develop a specific numeric strategy for determining whether such moment conditions can be constructed in particular models with discrete outcomes. In this section, we report the results from applying the approach in Honoré and Weidner (2022) to determine whether there are moments that can be used to identify and estimate $\rho$ in a Schmidt–Strauss model with lagged dependent variables and fixed effects.

We consider two versions of the model

$$\begin{aligned}{} & {} P\left( \left. y_{1,t}=c_{1},y_{2,t}=c_{2}\right| \left\{ y_{1,s},y_{2,s}\right\} _{s<t},\left\{ x_{1,s}\right\} _{s=1}^{T},\left\{ x_{2,s}\right\} _{s=1}^{T},\alpha _{1},\alpha _{2}\right) \\= & {} \frac{\exp \left( c_{1}\left( z_{1,t}+\alpha _{1}\right) +c_{2}\left( z_{2,t}+\alpha _{2}\right) +c_{1}c_{2}\rho \right) }{1+\exp \left( z_{1,t}+\alpha _{1}\right) +\exp (z_{2,t}+\alpha _{2})+\exp \left( z_{1,t}+\alpha _{1}+z_{2,t}+\alpha _{2}+\rho \right) } \end{aligned}$$

for $t=1,2,3$ and $$c_{1},c_{2}\in \left\{ 0,1\right\} \ $$, where $z_{1,t}=x_{1,t}^{\prime }\beta _{1}+y_{1,t-1}\gamma _{11}+y_{2,t-1}\gamma _{12}$ and $z_{2,t}=x_{2,t}^{\prime }\beta _{2}+y_{1,t-1}\gamma _{21}+y_{2,t-1}\gamma _{22}.$ In one version, $\alpha _{1}$ and $\alpha _{2}$ are unrestricted as in Section 5.1, while the other version restricts them to be identical except for an additive constant as in Section 5.3. Note that these are the same models as in Sects. 5.1 and 5.3, except that we here allow for strictly exogenous covariates.

Table 9 reports the number of moment conditions for each of the two versions of the model when one has 3, 4 or 5 time periods of observations in addition to the one that provides the initial conditions. The data used in this paper has a total of four consecutive time periods, and the results for $T=3$ are therefore the relevant ones here. In the empirical illustration in Sects. 5.2 and 5.4, we have no strictly exogenous time-varying explanatory variables, so according to the calculation reported in Table 9, there will be no moment conditions that depend on $\rho$ when the fixed effects are left unrestricted. On the other hand, there will be six moment conditions for each initial condition when the fixed effects are restricted. With more than three time periods (in addition to the one providing the initial conditions), the results suggest that there are moment conditions that depend on $\rho$ even when the fixed effects are unrestricted. While introducing explanatory variables changes the number of moment conditions, it does not change the answer to the question of whether there exist moment conditions that depend on $\rho$ for a given value of *T*.

**Table 9:** The number of moment conditions in the dynamic Schmidt–Strauss model with fixed effects

	$T=3$	$T=4$	$T=5$
$x_{k,t}=0$, unrestricted $(\alpha _{1},\alpha _{2})$	24 / 21 / 0	180 / 136 / 4	900 / 534 / 16
$x_{k,t}=0$, restricted $\alpha _{2} = \alpha _{1} + \kappa$	45 / 42 / 6	229 / 185 / 18	989 / 623 / 36
$x_{k,t} \ne 0$, unrestricted $(\alpha _{1},\alpha _{2})$	4 / 4 / 0	120 / 120 / 64	780 / 780 / 256
$x_{k,t} \ne 0$, restricted $\alpha _{2} = \alpha _{1} + \kappa$	45 / 45 / 16	229 / 229 / 48	989 / 989 / 96

Results from the numerical counting of moment conditions for the dynamic simultaneous logit are reported. Four different model specifications are considered: additional exogenous regressors are present ($x_{k,t} \ne 0$) or not ($x_{k,t} = 0$), and the fixed effects $(\alpha _{1},\alpha _{2})$ are unrestricted or restricted ($\alpha _{2} = \alpha _{1} + \kappa$). For each of those four specifications and each value of *T* we report $n_{\textrm{tot}} \, / \, n_{\textrm{para}} \, / \, n_{\rho }$, where $n_{\textrm{tot}}$ is the total number of moment conditions available, $n_{\textrm{para}}$ is the number of moment conditions available that depend on any of the common parameters ($\gamma _{11}$, $\gamma _{12}$, $\gamma _{21}$, $\gamma _{22}$ $\beta _1$, $\beta _2$, $\rho$, $\kappa$), and $n_{\rho }$ is the number of moment conditions available that depend on the parameter $\rho$. All results are for one fixed value of the initial condition $(y_{1,0},y_{2,0})$, but the number of moment conditions is independent from the initial condition. Notice that for $T=3$ and unrestricted $(\alpha _{1},\alpha _{2})$ we have $n_{\rho } = 0$, and in general we believe that the parameter is not identified in that case. However, for either $T>3$ or restricted $\alpha _{2} = \alpha _{1} + \kappa$ we find that $n_{\rho } > 0$ and the parameter $\rho$ can be identified and estimated from those moment conditions

### Moment conditions For $\rho$

It is not always easy to derive analytical expressions for the moment conditions. For the empirical application in Sects. 5.2 and 5.4 of this paper, *T* is three and there are no strictly exogenous time-varying explanatory variables. In order to make statements about $\rho$ , we therefore have to limit attention to the model in which the fixed effect is household specific in the sense that $\alpha _{2}=\alpha _{1}+\kappa$.

As mentioned above, there will be a total of 45 moment conditions in this case. One can write these as six that depend on $\rho$, 36 that depend on some of the common parameters in the model, but not on $\rho$, and three that do not depend on any of the parameters in the model. In principle, one may need to use all of these moments to construct an efficient GMM estimator. On the other hand, we can already identify the $\gamma$’s and $\kappa$ from the conditional likelihood approach in Sect. 5.3, so we only need to use one moment^8^ that depends on $\rho$ in order to (inefficiently) estimate $\rho$. We therefore focus on finding the six linearly independent moment conditions that depend on $\rho$. Unfortunately, these will not be unique. For example, adding a linear combination of moment conditions that do not depend on $\rho$ to one of the six that do, will leave us with six linearly independent moment conditions that depend on $\rho$. This also means that some of the moment conditions can be extremely complicated.

Fortunately, it turns out that for the model considered here, one can find six linearly independent moment conditions (for each initial condition) which all depend on $\rho$, and where each only depends on five of the 64 possible sequences. They are given in the Appendix, and we use those to estimate $\rho$ in the next subsection. These moment conditions are linear in $\exp \left( \rho \right)$.

### Empirical illustration

In this subsection, we illustrate how the method of moments approach discussed above can be used to estimate $\rho$ in the dynamic Schmidt–Strauss model with restricted fixed effects. We proceed in two steps. We first estimate the $\gamma$’s and $\kappa$ using the conditional likelihood approach. We then fix the $\gamma$’s and $\kappa$ at those estimates and estimate $\rho$ by generalized method of moments using the moment conditions in the Appendix. As weighting matrix, we use the inverse of a diagonal matrix that has the variance of the moments evaluated at $\rho =0$ in the diagonal. This choice is arbitrary and may lead to statistical inefficiency, but $\rho =0$ is a natural benchmark, and the hope is that using a diagonal matrix will alleviate small sample issues resulting from estimation of an efficient weighting matrix.^9^ Since the moment conditions are linear in $\exp \left( \rho \right)$, the GMM objective function will be quadratic in $\exp \left( \rho \right)$. This implies that it is numerically well behaved and that $\rho$ is actually identified from it. On the other hand, the solution for $\exp \left( \rho \right)$, can sometimes be negative in finite samples. For the estimation below, we search over values of $\rho$ between $-2$ and 4.

The results of the estimation of $\rho$ are presented in Table 10. Compared to the estimates of $\rho$ presented in Table 6 , the fixed effects estimates are much smaller. This suggests that the household specific fixed effect captures much more of the intra-household correlation than the observed characteristics.

**Table 10:** GMM estimation of $\rho$ by household ethnicity (restricted fixed effects)

	All	Whites	Blacks	Hispanics	Other	Mixed
$$\rho $$	1.260***	1.420***	0.360*	0.550***	0.730***	$$0.960^{***}$$
	(0.041)	(0.052)	(0.211)	(0.156)	(0.207)	(0.163)

***$$p<$$0.01, **$$p<$$0.05, *$$p<$$0.1

The dependent variable is working. The parameter $\rho$ is estimated by generalized method of moments using the moment conditions in the Appendix, and the $\gamma$’s and $\kappa$ by the conditional likelihood method in Sect. 5.3 . The data are from IPUMS CPS and cover a balanced panel of couples where each individual’s age is between 25 and 65. The data cover the period between 1982 and 2021. Standard errors are calculated via the bootstrap. Bootstrap estimates of the vector of $\gamma$’s are obtained by bootstrapping their influence function. Bootstrap estimates of $\rho$ are then calculated using GMM after recalculating the weighting matrix

**Fig. 9:**
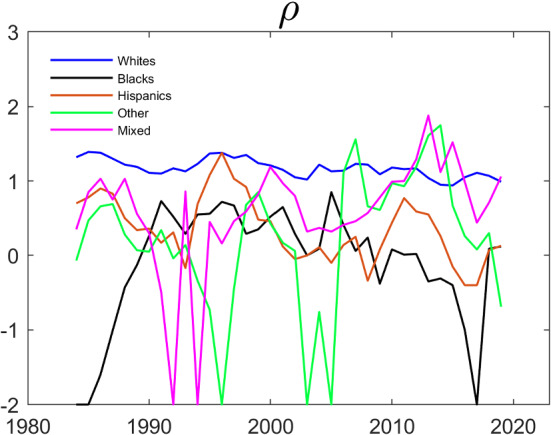
Evolution of $\rho$ over Time by Household Ethnicity (Restricted Fixed Effects). The dependent variable is working. $\rho$ is estimated by generalized method of moments using the moment conditions in the appendix, and the remaining parameters by the conditional likelihood method in Sect. 5.3 . The data are from IPUMS CPS and cover a balanced panel of couples where each individual’s age is between 25 and 65. The data cover the period between 1982 and 2021 and the estimation is done over five year centered rolling windows

Figure 9 presents the results of estimating $\rho$ separately for each ethnicity over rolling 5-year periods. The estimates for Whites seem fairly stable over time and are statistically significantly different from 0 in all time periods.^10^ When testing at a 5% level of significance, the estimates for the other ethnicities are statistically significantly different from 0 in only six of 144 cases (four for Blacks and two for Others).

## Dynamic Schmidt–Strauss models with correlated random effects

The calculations reported above establish that $\left( \gamma _{11},\gamma _{12},\gamma _{21},\gamma _{22},\kappa ,\rho \right)$ in the model in Sect. 5.3 is semiparametrically identified without assumptions on $\alpha$. In such cases, Wooldridge (2005) has proposed estimating $\left( \gamma _{11},\gamma _{12},\gamma _{21},\gamma _{22},\kappa ,\rho \right)$ by maximum likelihood conditional on the initial observations, $(y_{1,0},y_{2,0})$, after modeling the distribution of $\alpha$ conditional on those initial observations. This approach is in the spirit of Mundlak (1978) and Chamberlain (1982) and is known as a correlated random effects approach. See also Wooldridge (2019). If the conditional distribution of $\alpha$ given the initial conditions is sufficiently flexible, then one might interpret this approach as a semiparametric sieve maximum likelihood estimator.

Table 11 shows the estimates of $\left( \gamma _{11},\gamma _{12},\gamma _{21},\gamma _{22},\kappa ,\rho \right)$ that we obtain from the correlated random effects approach after modelling $\alpha$ conditional on $(y_{1,0},y_{2,0})$ as

10 $$\begin{aligned} \alpha =\delta _{0}+y_{1,0}\delta _{1}+y_{2,0}\delta _{2}+y_{1,0}y_{2,0}\delta _{3}+\nu ,\qquad \nu \sim N\left( 0,\sigma ^{2}\right) . \end{aligned}$$

**Table 11:** Estimates of dynamic Schmidt–Strauss model with correlated random effects by household ethnicity

	All	Whites	Blacks	Hispanics	Other	Mixed
$\gamma _{11}$	3.401***	3.414***	3.115***	3.297***	3.608***	3.405***
	(0.009)	(0.010)	(0.038)	(0.036)	(0.048)	(0.035)
$$\gamma _{12}$$	$$-2.686 $$***	$$-2.752 $$***	$$-2.274 $$***	$$-2.188 $$***	$$ -2.534 $$***	$$-2.629 $$***
	(0.010)	(0.011)	(0.045)	(0.042)	(0.057)	(0.041)
$$\gamma _{21}$$	$$-2.859 $$***	$$-2.933 $$***	$$-2.320 $$***	$$-2.404 $$***	$$ -2.654 $$***	$$-2.779 $$***
	(0.011)	(0.012)	(0.046)	(0.047)	(0.062)	(0.045)
$\gamma _{22}$	3.325***	3.383***	3.050***	2.898***	3.336***	3.278***
	(0.010)	(0.011)	(0.039)	(0.036)	(0.050)	(0.038)
$\rho$	0.866***	1.023***	0.013	0.007	0.496***	0.703***
	(0.011)	(0.012)	(0.050)	(0.048)	(0.066)	(0.045)
$\kappa$	0.855***	0.854***	0.354***	1.333***	0.894 ***	0.760***
	(0.007)	(0.008)	(0.027)	(0.027)	(0.035)	(0.029)

***$$p<$$0.01, **$$p<$$0.05, *$$p<$$0.1

The dependent variable is working and the parameters are estimated by maximizing the likelihood function conditional on the initial conditions and under the assumption that $\alpha$ is distributed as in Eq. (10). The data are from IPUMS CPS and cover a balanced panel of couples where each individual’s age is between 25 and 65. The data cover the period between 1982 and 2021

The estimates of $\left( \gamma _{11},\gamma _{12},\gamma _{21},\gamma _{22}\right)$ in Table 11 are larger in magnitude than those reported in Table 8, but the overall pattern is similar. The coefficients on one’s own past employment for women and for men, $\gamma _{11\text { }}$and $\gamma _{22}$, are positive and of the same magnitude, and the coefficients on the spouse’s past employment for women and for men, $\gamma _{12\text { }}$and $\gamma _{21}$, are negative and of the same magnitude. Moreover, these coefficients are estimated to be fairly similar across ethnicities. The estimates for $\rho$ in Table 11 show the same pattern as the estimates in Table 10. Whites have the largest coefficient, while the estimates for Blacks and Hispanics are much lower. The parameters estimated based on the correlated random effects approach have less sampling uncertainty than the fixed effects estimators in Sect. 5.3 (presumably because they are based on additional assumptions).

**Fig. 10:**
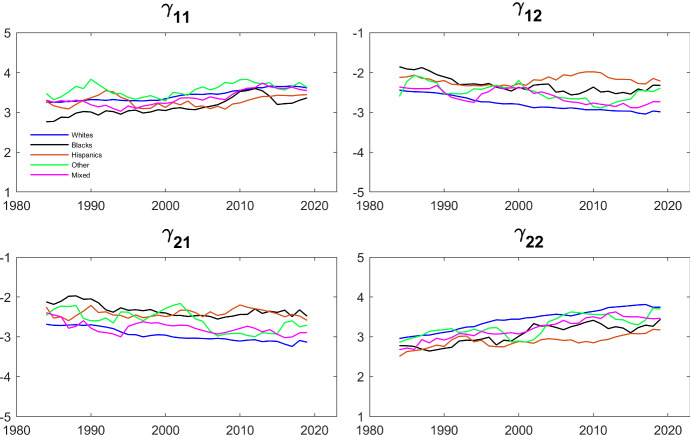
Evolution of $\gamma$’s over Time by Household Ethnicity (Correlated Random Effects). The dependent variable is working and the parameters are estimated by maximizing the conditional likelihood in Eq. (9). The data are from IPUMS CPS and cover a balanced panel of couples where each individual’s age is between 25 and 65. The data cover the period between 1982 and 2021 and the estimation is done over five year centered rolling windows

**Fig. 11:**
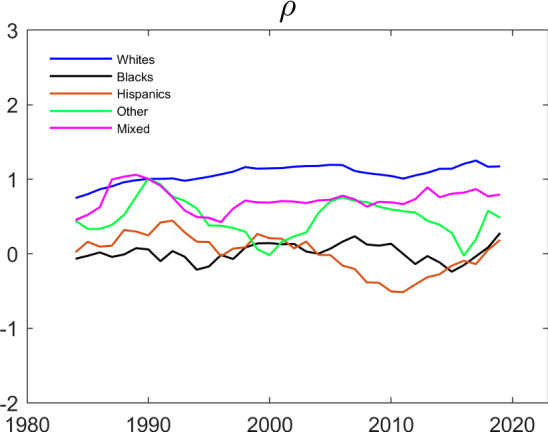
Evolution of $\rho$ over Time by Household Ethnicity (Correlated Random Effects). The dependent variable is working and the parameters are estimated by the correlated random effects approach. The data are from IPUMS CPS and cover a balanced panel of couples where each individual’s age is between 25 and 65. The data cover the period between 1982 and 2021 and the estimation is done over five year centered rolling windows

Figures 10 and 11 show the results of estimating the model using rolling 5-year sub-samples for each ethnicity. The estimates are fairly stable over time, and not very different across ethnicities. In terms of patterns, the results from estimating the $\gamma$ ’s presented in Fig. 10 mainly differ from the fixed effects estimates presented in Fig. 8 by displaying a clearer upward trend in the husband’s coefficient on his own past employment, $\gamma _{22}$. The estimates also tend to have less sampling uncertainty. Again, this is to be expected because the correlated random effects approach imposes additional structure relative to the fixed effects approach. The correlated random effects estimates of the $\rho$’s presented in Fig. 11 are also noticeably less volatile than the GMM estimates in Fig. 9.

The fact that the correlated random effects approach is associated with less sampling uncertainty than the conditional likelihood approach comes at a price: If the parametric form for the individual specific effect is misspecified then the estimator can be inconsistent. For a given simple data generating process, one can gauge the importance of this by calculating the maximizer of the limiting (the expected) log-likelihood function for the conditional random effects model. This is especially easy if the data generating process for the fixed effects is discrete because the limiting objective function becomes a sum rather than an integral in that case. This maximizer of the limiting log-likelihood function will be the probability limit of the conditional random effects estimator. To illustrate this, let $$ \left( \gamma _{11},\gamma _{12},\gamma _{21},\gamma _{22},\rho ,\kappa \right) =\left( 2.5,-1.5,-1.5,2.5,1,2\right) $$ and assume that $$y_{1,0}$$ and $$y_{2,0} $$ are independent and equal to 1 with probability $$\frac{1}{2}$$. We can then maximize the limiting objective functions for the correlated random effects that assumes (10) under the following distributions for $$ \alpha $$:

- Correctly specified: $\alpha =-1+y_{1,0}+y_{2,0}+\nu$, where $\nu \sim N\left( 0,1\right) .$
- Discrete, but approximately normal: $\alpha =\eta$ where
  - $$P\left( \left. \eta =-d\right| y_{1,0},y_{2,0}\right) =P\left( \left. \eta =d\right| y_{1,0},y_{2,0}\right) =\varPhi \left( -1.5\right) ,$$
  - $$P\left( \left. \eta =-1\right| y_{1,0},y_{2,0}\right) =P\left( \left. \eta =1\right| y_{1,0},y_{2,0}\right) =\varPhi \left( 1.5\right) -\varPhi \left( 0.5\right) $$, and
  - $$P\left( \left. \eta =0\right| y_{1,0},y_{2,0}\right) =\varPhi \left( 0.5\right) -\varPhi \left( -0.5\right) $$,
  where $\varPhi$ is the standard normal cumulative distribution function and $d\approx 1.9662$ is chosen such that $\eta$ has variance 1.
- Discrete, asymmetric: $$P\left( \left. \alpha =3\right| y_{1,0},y_{2,0}\right) =\frac{1}{4}$$, $$P\left( \left. \alpha =-1\right| y_{1,0},y_{2,0}\right) =\frac{3}{4}$$.
- Heteroskedastic: $$P\left( \left. \alpha =-\sqrt{2+2y_{1,0}}\right| y_{1,0},y_{2,0}\right) =P\big ( \left. \alpha =\sqrt{2+2y_{1,0}}\right| y_{1,0},y_{2,0}\big ) =\frac{1}{2}$$.
- Very heteroskedastic:$$P\left( \left. \alpha =-\sqrt{5y_{1,0}} \right| y_{1,0},y_{2,0}\right) =P\left( \left. \alpha =\sqrt{5y_{1,0}} \right| y_{1,0},y_{2,0}\right) =\frac{1}{2}$$.

The results are in Table 12.

**Table 12:** Probability limit of conditional random effects estimator under different heterogeneity distributions

Distribution of heterogeneity	$\gamma _{11}$	$\gamma _{12}$	$\gamma _{21}$	$\gamma _{22}$	$\rho$	$\kappa$
Correctly specified	2.50	$-1.50$	$-1.50$	2.50	1.00	2.00
Discrete, but approximately normal	2.51	$-1.50$	$-1.52$	2.49	0.99	2.02
Discrete, asymmetric	2.66	$-1.73$	$-1.61$	2.42	0.95	2.08
Heteroskedastic	2.68	$-1.62$	$-1.92$	2.44	1.00	2.39
Very heteroskedastic	2.64	$$-1.29 $$	$$-1.91$$	2.63	1.27	2.53

The table gives the probability limit of the correlated random effects estimator for various distributions of the fixed effect when $\left( \gamma _{11},\gamma _{12},\gamma _{21},\gamma _{22},\rho ,\kappa \right) =\left( 2.5,-1.5,-1.5,2.5,1,2\right)$ and $y_{1,0}$ and $y_{2,0}$ are independent and equal to 1 with probability $\frac{1}{2}$

The probability limits in Table 12 illustrate that the correlated random effects approach can provide a very good approximation when the distribution of the heterogeneity ($\alpha$) is well-approximated by the assumed functional form, but also that the biases can be a much larger source of estimation error for the estimator than sampling variance for the kind of sample sizes considered here.

## Conclusion

Two of Peter Schmidt’s many contributions to econometrics have been to introduce an econometric model for simultaneous binary outcomes and to study the estimation of dynamic linear fixed effects panel data models using short panels. In this paper, we combine aspects of this research by studying panel data versions of the model introduced in Schmidt and Strauss (1975) that allow for lagged dependent variables and fixed effects, and we apply existing as well as new methods to investigate the joint behavior of employment of husbands and wives.

On the methodological side, we first use the conditional likelihood approach of Honoré and Kyriazidou (2019) to construct a likelihood function that does not depend on the fixed effects of the model. While this conditional likelihood can be used to estimate the other parameters of the model when the total number of time periods is at least four, it turns out that it does not depend on the parameter $\rho$, which in the Schmidt–Strauss model captures the inter-equation dependence. As a result, our conditional likelihood approach cannot be used to estimate this parameter. We therefore next use the approach in Honoré and Weidner (2022) to study whether one can construct moment conditions that can be used to estimate $\rho$. We find that it is in principle possible to estimate the common parameters of such models when the total number of time periods for each individual is at least five. To construct moment conditions for four time periods, it is necessary to restrict the model. We do this by restricting the fixed effects for the two outcomes to be equal, except for an additive constant.

On the empirical side, we apply existing methods like those developed in Schmidt and Strauss (1975), as well as the estimation methods developed in this paper, to estimate a simple model for the relationship of employment of husbands and wives. Our main conclusion is that the parameter that captures the intra-household dependence in employment varies by the ethnicity composition of the couple and over time, even after one allows for unobserved household specific heterogeneity.

## Appendix: Moment conditions

In this Appendix, we explicitly present the six moment conditions discussed in Sect. 6.1. To simplify the notation, we write $\varGamma _{ij}=\exp \left( \gamma _{ij}\right)$, $B=\exp \left( \beta \right)$, and $P=\exp \left( \rho \right)$.

### Moment condition 1

$$\begin{aligned} E\left[ \sum \limits _{k=1}^{5}m_{k}1\left\{ \left( \left\{ y_{1,t}\right\} _{t=0}^{3},\left\{ y_{2,t}\right\} _{t=0}^{3}\right) =s_{k}\right\} \right] =0 \end{aligned}$$

where

$$\begin{aligned} s_{1}= & {} (a,0,0,1,b,0,1,0) \\ s_{2}= & {} (a,0,0,1,b,0,1,1) \\ s_{3}= & {} (a,0,1,0,b,0,1,0) \\ s_{4}= & {} (a,0,1,0,b,1,1,0) \\ s_{5}= & {} (a,0,1,1,b,0,1,0) \end{aligned}$$

and

$$\begin{aligned}{} & {} m_{1}=B\varGamma _{11}\varGamma _{22}P\Bigl [\varGamma _{12}\left( -B\varGamma _{21}\varGamma _{22}^{2}+(B+1)\varGamma _{22}+\varGamma _{11}\left( \varGamma _{21}\varGamma _{22}\left( B\varGamma _{22}-B-1\right) +1\right) -1\right) \Bigr . \\{} & {} \quad \qquad +\Bigl .B\left( \varGamma _{21}-1\right) \varGamma _{22}+\varGamma _{11}\left( \varGamma _{21}-1\right) \varGamma _{22}\varGamma _{12}^{2}\Bigr ]\\{} & {} m_{2}=\varGamma _{11}\Bigl [B^{2}\left( \varGamma _{21}-1\right) \varGamma _{22}^{2}+ \\{} & {} \qquad \varGamma _{12}^{2}\left( -B\varGamma _{22}^{2}P+\varGamma _{11}\left( B\varGamma _{21}\varGamma _{22}^{2}P-(B+1)\varGamma _{22}+1\right) +(B+1)\varGamma _{22}-1\right) \Bigr . \\{} & {} \qquad +\Bigl .B\varGamma _{22}\varGamma _{12}\left( B\varGamma _{22}-\varGamma _{21}\left( (B+1)\varGamma _{22}-1\right) +\varGamma _{11}\left( 1-\varGamma _{21}P\right) +\varGamma _{22}+P-2\right) \Bigr ]\\{} & {} m_{3}=-B\varGamma _{11}\varGamma _{12}\varGamma _{22} \\{} & {} \qquad \Bigl [\varGamma _{12}\left( -B\varGamma _{21}\varGamma _{22}^{2}+(B+1)\varGamma _{22}+\varGamma _{11}\left( \varGamma _{21}\varGamma _{22}\left( B\varGamma _{22}-B-1\right) +1\right) -1\right) \Bigr . \\{} & {} \qquad +\Bigl .B\left( \varGamma _{21}-1\right) \varGamma _{22}+\varGamma _{11}\left( \varGamma _{21}-1\right) \varGamma _{22}\varGamma _{12}^{2}\Bigr ]\\{} & {} m_{4}=-\varGamma _{11}\varGamma _{12}\varGamma _{21}^{-a}\varGamma _{22}^{1-b}\Bigl [ \varGamma _{11} \\{} & {} \left( B^{2}\left( \varGamma _{21}-1\right) \varGamma _{21}\varGamma _{22}^{2}+B\varGamma _{22}\varGamma _{12}\left( 2\varGamma _{21}(P-2)+\varGamma _{21}^{2}+1\right) -\left( \left( \varGamma _{21}-1\right) \varGamma _{12}^{2}\right) \right) \Bigr . \\{} & {} \qquad +\Bigl .\varGamma _{12}\varGamma _{11}^{2}\left( B\varGamma _{21}\varGamma _{22}\left( 1-\varGamma _{21}P\right) +\varGamma _{12}\left( \varGamma _{21}-1\right) \right) +B\varGamma _{22}\left( \varGamma _{12}\left( \varGamma _{21}-P\right) -B\left( \varGamma _{21}-1\right) \varGamma _{21}\varGamma _{22}\right) \Bigr ]\\{} & {} m_{5}=B\varGamma _{22}\Bigl [\varGamma _{11}^{2}\varGamma _{12}^{2}\left( -B\varGamma _{21}^{2}\varGamma _{22}^{2}P+(B+1)\varGamma _{21}\varGamma _{22}-1\right) \Bigr . \\{} & {} \qquad +\varGamma _{11}\varGamma _{12}\bigl ( B\varGamma _{22}\left( \varGamma _{21}\left( -(B+1)\varGamma _{22}+P-2\right) +(B+1)\varGamma _{22}\varGamma _{21}^{2}+1\right) \\{} & {} \qquad +\varGamma _{12}\left( \varGamma _{21}\varGamma _{22}\left( B\varGamma _{22}P-B-1\right) +1\right) \bigr ) \\{} & {} \qquad +\Bigl .B\varGamma _{22}\left( \varGamma _{12}\left( \varGamma _{21}-P\right) -B\left( \varGamma _{21}-1\right) \varGamma _{21}\varGamma _{22}\right) \Bigr ] \end{aligned}$$

### Moment condition 2

$$\begin{aligned} E\left[ \sum \limits _{k=1}^{5}m_{k}1\left\{ \left( \left\{ y_{1,t}\right\} _{t=0}^{3},\left\{ y_{2,t}\right\} _{t=0}^{3}\right) =s_{k}\right\} \right] =0 \end{aligned}$$

where

$$\begin{aligned} s_{1}= & {} (a,0,0,1,b,1,0,0) \\ s_{2}= & {} (a,0,0,1,b,1,0,1) \\ s_{3}= & {} (a,0,1,0,b,0,1,1) \\ s_{4}= & {} (a,1,0,0,b,1,0,0) \\ s_{5}= & {} (a,1,0,0,b,1,1,0) \end{aligned}$$

and

$$\begin{aligned} m_{1}= & {} -B\varGamma _{21}P\varGamma _{11}^{a}\varGamma _{12}^{b}\left[ \varGamma _{12}\left( -B\varGamma _{21}\varGamma _{22}^{2}+(B+1)\varGamma _{22}+\varGamma _{11}\left( \varGamma _{21}\varGamma _{22}\left( B\varGamma _{22}-B-1\right) +1\right) -1\right) \right. \\{} & {} +\left. B\left( \varGamma _{21}-1\right) \varGamma _{22}+\varGamma _{11}\left( \varGamma _{21}-1\right) \varGamma _{22}\varGamma _{12}^{2}\right] \\ m_{2}= & {} \varGamma _{21}\left( -\varGamma _{11}^{a}\right) \varGamma _{12}^{b}\left[ B^{2}\left( \varGamma _{21}-1\right) \varGamma _{22}^{2}\right. \\{} & {} +\varGamma _{12}^{2}\left( -B\varGamma _{22}^{2}P+\varGamma _{11}\left( B\varGamma _{21}\varGamma _{22}^{2}P-(B+1)\varGamma _{22}+1\right) +(B+1)\varGamma _{22}-1\right) \\{} & {} +\left. B\varGamma _{22}\varGamma _{12}\left( B\varGamma _{22}-\varGamma _{21}\left( (B+1)\varGamma _{22}-1\right) +\varGamma _{11}\left( 1-\varGamma _{21}P\right) +\varGamma _{22}+P-2\right) \right] \\ m_{3}= & {} \varGamma _{11}^{a}\varGamma _{21}^{a}\varGamma _{12}^{b}\varGamma _{22}^{b-1} \left[ \varGamma _{11}^{2}\varGamma _{12}^{2}\left( B\varGamma _{21}^{2}\varGamma _{22}^{2}P-(B+1)\varGamma _{21}\varGamma _{22}+1\right) \right. \\{} & {} -\varGamma _{11}\varGamma _{12}\bigl ( \ B\varGamma _{22}\left( \varGamma _{21}\left( -(B+1)\varGamma _{22}+P-2\right) +(B+1)\varGamma _{22}\varGamma _{21}^{2}+1\right) \\{} & {} +\varGamma _{12}\left( \varGamma _{21}\varGamma _{22}\left( B\varGamma _{22}P-B-1\right) +1\right) \bigr ) \\{} & {} +\left. B\varGamma _{22}\left( B\left( \varGamma _{21}-1\right) \varGamma _{21}\varGamma _{22}+\varGamma _{12}\left( P-\varGamma _{21}\right) \right) \right] \\ m_{4}= & {} B\varGamma _{21}\left[ \varGamma _{12}\left( -B\varGamma _{21}\varGamma _{22}^{2}+(B+1)\varGamma _{22}+\varGamma _{11}\left( \varGamma _{21}\varGamma _{22}\left( B\varGamma _{22}-B-1\right) +1\right) -1\right) \right. \\{} & {} +\left. B\left( \varGamma _{21}-1\right) \varGamma _{22}+\varGamma _{11}\left( \varGamma _{21}-1\right) \varGamma _{22}\varGamma _{12}^{2}\right] \\ m_{5}= & {} \varGamma _{12}\left[ \varGamma _{11}\left( B^{2}\left( \varGamma _{21}-1\right) \varGamma _{21}\varGamma _{22}^{2}+B\varGamma _{22}\varGamma _{12}\left( 2\varGamma _{21}(P-2)+\varGamma _{21}^{2}+1\right) -\left( \left( \varGamma _{21}-1\right) \varGamma _{12}^{2}\right) \right) \right. + \\{} & {} \left. \varGamma _{12}\varGamma _{11}^{2}\left( B\varGamma _{21}\varGamma _{22}\left( 1-\varGamma _{21}P\right) +\varGamma _{12}\left( \varGamma _{21}-1\right) \right) +B\varGamma _{22}\left( \varGamma _{12}\left( \varGamma _{21}-P\right) -B\left( \varGamma _{21}-1\right) \varGamma _{21}\varGamma _{22}\right) \right] \end{aligned}$$

### Moment condition 3

$$\begin{aligned} E\left[ \sum \limits _{k=1}^{5}m_{k}1\left\{ \left( \left\{ y_{1,t}\right\} _{t=0}^{3},\left\{ y_{2,t}\right\} _{t=0}^{3}\right) =s_{k}\right\} \right] =0 \end{aligned}$$

where

$$\begin{aligned} s_{1}= & {} (a,0,1,1,b,1,0,1) \\ s_{2}= & {} (a,0,1,1,b,1,1,1) \\ s_{3}= & {} (a,1,0,0,b,1,1,1) \\ s_{4}= & {} (a,1,1,1,b,0,1,0) \\ s_{5}= & {} (a,1,1,1,b,0,1,1) \end{aligned}$$

and

$$\begin{aligned} m_{1}= & {} -B\varGamma _{11}^{a+1}\varGamma _{21}^{-a}\varGamma _{12}^{b}\varGamma _{22}^{1-b} \\{} & {} \left[ \varGamma _{11}(B^{2}\varGamma _{21}\left( \varGamma _{21}-\varGamma _{22}\right) \varGamma _{22}\right. \\{} & {} +B\varGamma _{12}\left( 2\varGamma _{22}\varGamma _{21}(P-2)+\varGamma _{21}^{2}+\varGamma _{22}^{2}\right) +\varGamma _{12}^{2}\left( \varGamma _{22}-\varGamma _{21}\right) ) \\{} & {} +\left. \varGamma _{11}^{2}\left( B\varGamma _{21}\left( \varGamma _{22}-\varGamma _{21}P\right) +\varGamma _{12}\left( \varGamma _{21}-\varGamma _{22}\right) \right) +B\varGamma _{12}\varGamma _{22}\left( B\varGamma _{21}\left( \varGamma _{22}-\varGamma _{21}\right) +\varGamma _{12}\left( \varGamma _{21}-\varGamma _{22}P\right) \right) \right] \\ m_{2}= & {} -B\varGamma _{11}^{a+1}\varGamma _{21}^{-a}\varGamma _{12}^{b}\varGamma _{22}^{-b} \\{} & {} \left[ \varGamma _{11}\left( \left( \varGamma _{21}-\varGamma _{22}\right) \varGamma _{12}\left( B\varGamma _{21}\varGamma _{22}+1\right) -B\left( \varGamma _{21}-1\right) \varGamma _{21}\varGamma _{22}-\left( \left( \varGamma _{21}-1\right) \varGamma _{22}\varGamma _{12}^{2}\right) \right) \right. \\{} & {} +\left. B\varGamma _{12}\varGamma _{21}\left( \varGamma _{22}-1\right) \varGamma _{22}+\varGamma _{12}\varGamma _{21}\left( \varGamma _{22}-1\right) \varGamma _{11}^{2} \right] \\ m_{3}= & {} \varGamma _{11}^{2}\varGamma _{12}\varGamma _{21}^{-a}\varGamma _{22}^{-b-1} \\{} & {} \bigl [ \varGamma _{11}(B^{2}\left( \varGamma _{21}-1\right) \varGamma _{21}\varGamma _{22}^{2}+\varGamma _{12}^{2}\left( \varGamma _{21}\left( B\varGamma _{22}^{2}P-B\varGamma _{22}-1\right) +\varGamma _{22}\right) \\{} & {} +B\varGamma _{22}\varGamma _{12}\left( \varGamma _{21}\left( -\varGamma _{22}+P-2\right) +\varGamma _{21}^{2}+\varGamma _{22}\right) ) \\{} & {} +\varGamma _{12}\varGamma _{11}^{2}\left( B\varGamma _{21}\varGamma _{22}\left( 1-\varGamma _{21}P\right) +\varGamma _{12}\left( \varGamma _{21}-\varGamma _{22}\right) \right) \\{} & {} +B\varGamma _{12}\varGamma _{22}\left( \varGamma _{12}\left( \varGamma _{21}-\varGamma _{22}P\right) -B\left( \varGamma _{21}-1\right) \varGamma _{21}\varGamma _{22}\right) \bigr ]\\ m_{4}= & {} B^{2}\varGamma _{22}\bigl [ B^{2}\varGamma _{12}\varGamma _{21}^{2}\left( \varGamma _{22}-1\right) \varGamma _{22}+ \\{} & {} \varGamma _{11}^{2}\left( \varGamma _{12}\left( B\varGamma _{22}\varGamma _{21}^{2}P+\varGamma _{21}\left( 1-B\varGamma _{22}\right) -\varGamma _{22}\right) +B\varGamma _{21}\left( \varGamma _{22}-\varGamma _{21}P\right) +\left( \varGamma _{22}-\varGamma _{21}\right) \varGamma _{12}^{2}\right) \\{} & {} +B\varGamma _{21}\varGamma _{11}\left( -B\varGamma _{21}\left( \varGamma _{22}-1\right) \varGamma _{22}+\varGamma _{12}^{2}\left( \varGamma _{22}-\varGamma _{22}^{2}P\right) +\varGamma _{12}\left( \varGamma _{22}\left( \varGamma _{22}+P-2\right) -\varGamma _{21}\left( \varGamma _{22}-1\right) \right) \right) \bigr ]\\ m_{5}= & {} B^{2}\varGamma _{11}\left[ \varGamma _{11}\left( \left( \varGamma _{21}-\varGamma _{22}\right) \varGamma _{12}\left( B\varGamma _{21}\varGamma _{22}+1\right) -B\left( \varGamma _{21}-1\right) \varGamma _{21}\varGamma _{22}-\left( \left( \varGamma _{21}-1\right) \varGamma _{22}\varGamma _{12}^{2}\right) \right) \right. \\{} & {} +\left. B\varGamma _{12}\varGamma _{21}\left( \varGamma _{22}-1\right) \varGamma _{22}+\varGamma _{12}\varGamma _{21}\left( \varGamma _{22}-1\right) \varGamma _{11}^{2} \right] \end{aligned}$$

### Moment condition 4

$$\begin{aligned} E\left[ \sum \limits _{k=1}^{5}m_{k}1\left\{ \left( \left\{ y_{1,t}\right\} _{t=0}^{3},\left\{ y_{2,t}\right\} _{t=0}^{3}\right) =s_{k}\right\} \right] =0 \end{aligned}$$

where

$$\begin{aligned} s_{1}= & {} (a,1,0,0,b,1,0,1) \\ s_{2}= & {} (a,1,0,1,b,0,0,1) \\ s_{3}= & {} (a,1,0,1,b,1,0,1) \\ s_{4}= & {} (a,1,1,0,b,1,0,0) \\ s_{5}= & {} (a,1,1,0,b,1,0,1) \end{aligned}$$

and

$$\begin{aligned} m_{1}= & {} \varGamma _{12} \\{} & {} \left[ \varGamma _{11}\left( -\varGamma _{21}\left( B^{2}+\varGamma _{12}\left( B-B\varGamma _{22}P\right) +B\varGamma _{22}-BP+2B+1\right) +B(B+1)\varGamma _{22}\varGamma _{21}^{2}+B+1\right) \right. \\{} & {} +\left. \varGamma _{12}\varGamma _{11}^{2}\left( -B\varGamma _{22}\varGamma _{21}^{2}P+(B+1)\varGamma _{21}-1\right) +B\left( -B\varGamma _{22}\varGamma _{21}^{2}+(B+1)\varGamma _{21}-P\right) \right] \\ m_{2}= & {} -B\varGamma _{12}\varGamma _{21}^{a}\varGamma _{22}^{b}\left[ \varGamma _{11}\left( -\varGamma _{21}\left( B^{2}-2BP+4B+1\right) +B(B+1)\varGamma _{21}^{2}+B+1\right) \right. \\{} & {} +\left. \varGamma _{11}^{2}\left( -B\varGamma _{21}^{2}P+(B+1)\varGamma _{21}-1\right) +B\left( -B\varGamma _{21}^{2}+(B+1)\varGamma _{21}-P\right) \right] \\ m_{3}= & {} -B\varGamma _{12}\left[ \varGamma _{11}\left( -B\varGamma _{22}\varGamma _{21}^{2}+(B+1)\varGamma _{21}+\varGamma _{12}\left( B\varGamma _{22}\varGamma _{21}^{2}-(B+1)\varGamma _{22}\varGamma _{21}+1\right) -1\right) \right. \\{} & {} +\left. B\varGamma _{21}\left( \varGamma _{22}-1\right) +\varGamma _{12}\varGamma _{21}\left( \varGamma _{22}-1\right) \varGamma _{11}^{2}\right] \\ m_{4}= & {} B\left[ B^{2}\left( \varGamma _{22}-1\right) \varGamma _{21}^{2}\varGamma _{11}^{-1}\right. \\{} & {} +B\varGamma _{21}\left( -(B+1)\varGamma _{21}\left( \varGamma _{22}-1\right) +\varGamma _{12}\left( 1-\varGamma _{22}P\right) +\varGamma _{22}+P-2\right) \\{} & {} +\left. \varGamma _{11}\left( -B\varGamma _{21}^{2}P+\varGamma _{12}\left( B\varGamma _{22}\varGamma _{21}^{2}P-(B+1)\varGamma _{21}+1\right) +(B+1)\varGamma _{21}-1\right) \right] \\ m_{5}= & {} BP\left[ \varGamma _{11}\left( -B\varGamma _{22}\varGamma _{21}^{2}+(B+1)\varGamma _{21}+\varGamma _{12}\left( B\varGamma _{22}\varGamma _{21}^{2}-(B+1)\varGamma _{22}\varGamma _{21}+1\right) -1\right) \right. \\{} & {} +\left. B\varGamma _{21}\left( \varGamma _{22}-1\right) +\varGamma _{12}\varGamma _{21}\left( \varGamma _{22}-1\right) \varGamma _{11}^{2}\right] \end{aligned}$$

### Moment condition 5

$$\begin{aligned} E\left[ \sum \limits _{k=1}^{5}m_{k}1\left\{ \left( \left\{ y_{1,t}\right\} _{t=0}^{3},\left\{ y_{2,t}\right\} _{t=0}^{3}\right) =s_{k}\right\} \right] =0 \end{aligned}$$

where

$$\begin{aligned} s_{1}= & {} (a,0,0,0,b,0,1,0) \\ s_{2}= & {} (a,0,0,0,b,0,1,1) \\ s_{3}= & {} (a,0,1,0,b,0,0,0) \\ s_{4}= & {} (a,0,1,0,b,0,0,1) \\ s_{5}= & {} (a,0,1,0,b,1,0,0) \end{aligned}$$

and

$$\begin{aligned} m_{1}= & {} B\varGamma _{12}\varGamma _{21}^{a}\varGamma _{22}^{b}\left[ B\left( \varGamma _{22}-\varGamma _{21}\right) +\varGamma _{12}\left( \varGamma _{22}\left( B\varGamma _{21}-B-1\right) +1\right) \right. \\{} & {} +\left. \varGamma _{11}\left( \varGamma _{21}\left( B\left( -\varGamma _{22}\right) +B-\varGamma _{12}+1\right) +\varGamma _{12}\varGamma _{22}-1\right) \right] \\ m_{2}= & {} \varGamma _{12}\varGamma _{21}^{a}\varGamma _{22}^{b-1} \\{} & {} \left[ B^{2}\varGamma _{22}\left( \varGamma _{22}-\varGamma _{21}\right) +B\varGamma _{12}\left( \varGamma _{21}\left( (B+1)\varGamma _{22}-1\right) -\varGamma _{22}\left( (B+1)\varGamma _{22}+P-2\right) \right) \right. \\{} & {} +\varGamma _{12}^{2}\left( B\varGamma _{22}^{2}P-(B+1)\varGamma _{22}+1\right) \\{} & {} \left. +\varGamma _{11}\left( B\left( \varGamma _{21}P-\varGamma _{22}\right) +\varGamma _{12}\left( \varGamma _{22}\left( B\varGamma _{21}(-P)+B+1\right) -1\right) \right) \right] \\ m_{3}= & {} -B^{2}\varGamma _{12}\varGamma _{21}^{a}\varGamma _{22}^{b}\left[ B\left( \varGamma _{22}-\varGamma _{21}\right) +\varGamma _{12}\left( \varGamma _{22}\left( B\varGamma _{21}-B-1\right) +1\right) \right. \\{} & {} +\left. \varGamma _{11}\left( \varGamma _{21}\left( B\left( -\varGamma _{22}\right) +B-\varGamma _{12}+1\right) +\varGamma _{12}\varGamma _{22}-1\right) \right] \\ m_{4}= & {} -B\varGamma _{12}\varGamma _{21}^{a-1}\varGamma _{22}^{b}\left[ \varGamma _{11}^{2}\left( -B\varGamma _{21}^{2}P+(B+1)\varGamma _{21}-1\right) \right. \\{} & {} +\varGamma _{11}\bigl [ B\left( \varGamma _{21}\left( -(B+1)\varGamma _{22}+P-2\right) +(B+1)\varGamma _{21}^{2}+\varGamma _{22}\right) \\{} & {} +\varGamma _{12}\left( \varGamma _{21}\left( B\varGamma _{22}P-B-1\right) +1\right) \bigr ] \\{} & {} +\left. B\left( B\varGamma _{21}\left( \varGamma _{22}-\varGamma _{21}\right) +\varGamma _{12}\left( \varGamma _{21}-\varGamma _{22}P\right) \right) \right] \\ m_{5}= & {} B\left[ \varGamma _{11}\left( B^{2}\varGamma _{21}\left( \varGamma _{21}-\varGamma _{22}\right) \varGamma _{22}+B\varGamma _{12}\left( 2\varGamma _{22}\varGamma _{21}(P-2)+\varGamma _{21}^{2}+\varGamma _{22}^{2}\right) +\varGamma _{12}^{2}\left( \varGamma _{22}-\varGamma _{21}\right) \right) \right. \\{} & {} +\left. \varGamma _{11}^{2}\left( B\varGamma _{21}\left( \varGamma _{22}-\varGamma _{21}P\right) +\varGamma _{12}\left( \varGamma _{21}-\varGamma _{22}\right) \right) +B\varGamma _{12}\varGamma _{22}\left( B\varGamma _{21}\left( \varGamma _{22}-\varGamma _{21}\right) +\varGamma _{12}\left( \varGamma _{21}-\varGamma _{22}P\right) \right) \right] \end{aligned}$$

### Moment condition 6

$$\begin{aligned} E\left[ \sum \limits _{k=1}^{5}m_{k}1\left\{ \left( \left\{ y_{1,t}\right\} _{t=0}^{3},\left\{ y_{2,t}\right\} _{t=0}^{3}\right) =s_{k}\right\} \right] =0 \end{aligned}$$

where

$$\begin{aligned} s_{1}= & {} (a,0,0,0,b,1,0,1) \\ s_{2}= & {} (a,0,1,0,b,1,0,1) \\ s_{3}= & {} (a,0,1,1,b,0,0,0) \\ s_{4}= & {} (a,0,1,1,b,0,0,1) \\ s_{5}= & {} (a,1,0,0,b,0,1,0) \end{aligned}$$

and

$$\begin{aligned} m_{1}= & {} \varGamma _{11}^{a}\varGamma _{21}^{1-a}\varGamma _{12}^{b}\varGamma _{22}^{-b} \left[ -\varGamma _{12}\left( -\varGamma _{22}\left( B^{2}-2BP+4B+1\right) +B(B+1)\varGamma _{22}^{2}+B+1\right) \right. \\{} & {} +\left. \varGamma _{12}^{2}\left( B\varGamma _{22}^{2}P-(B+1)\varGamma _{22}+1\right) +B\left( B\varGamma _{22}^{2}-(B+1)\varGamma _{22}+P\right) \right] \\ m_{2}= & {} BP\varGamma _{11}^{a}\varGamma _{21}^{-a}\varGamma _{12}^{b}\varGamma _{22}^{1-b} \left[ B\left( \varGamma _{21}-\varGamma _{22}\right) +\varGamma _{12}\left( \varGamma _{22}\left( B\left( -\varGamma _{21}\right) +B+1\right) -1\right) \right. \\{} & {} +\left. \varGamma _{11}\left( \varGamma _{21}\left( B\varGamma _{22}-B+\varGamma _{12}-1\right) -\varGamma _{12}\varGamma _{22}+1\right) \right] \\ m_{3}= & {} B^{2}\varGamma _{21}\varGamma _{11}^{a-1}\varGamma _{12}^{b} \\{} & {} \bigl [ \varGamma _{12}(-B^{2}\varGamma _{22}+B\varGamma _{22}P+\varGamma _{11}\left( \varGamma _{22}\left( B\varGamma _{21}(-P)+B+1\right) -1\right) \\{} & {} -2B\varGamma _{22}+B\varGamma _{21}\left( (B+1)\varGamma _{22}-1\right) +B-\varGamma _{22}+1) \\{} & {} +B\left( \varGamma _{22}\left( B\left( -\varGamma _{21}\right) +B+1\right) +\varGamma _{11}\left( \varGamma _{21}P-\varGamma _{22}\right) -P\right) \bigr ]\\ m_{4}= & {} B^{2}\varGamma _{11}^{a-1}\varGamma _{12}^{b}\left[ B\left( \varGamma _{22}-\varGamma _{21}\right) +\varGamma _{12}\left( \varGamma _{22}\left( B\varGamma _{21}-B-1\right) +1\right) \right. \\{} & {} +\left. \varGamma _{11}\left( \varGamma _{21}\left( B\left( -\varGamma _{22}\right) +B-\varGamma _{12}+1\right) +\varGamma _{12}\varGamma _{22}-1\right) \right] \\ m_{5}= & {} B\left[ B^{2}\left( \varGamma _{21}-\varGamma _{22}\right) \varGamma _{22}+B\varGamma _{12}\left( \varGamma _{22}\left( (B+1)\varGamma _{22}+P-2\right) -\varGamma _{21}\left( (B+1)\varGamma _{22}-1\right) \right) \right. \\{} & {} +\varGamma _{12}^{2}\left( -B\varGamma _{22}^{2}P+(B+1)\varGamma _{22}-1\right) \\{} & {} +\left. \varGamma _{11}\left( B\left( \varGamma _{22}-\varGamma _{21}P\right) +\varGamma _{12}\left( \varGamma _{22}\left( B\varGamma _{21}P-B-1\right) +1\right) \right) \right] \end{aligned}$$

## Footnotes

1 The reason why we focus on the case where the two probabilities are equal is that different values of the probabilities will bound the correlation away from $-1$ or 1.

2 We further clean the data by eliminating individuals with missing or logically inconsistent age increases from one period to the next or inconsistent sex or race over time.

3 The 2008 financial crisis and the onset of the pandemic in 2020 are possible exceptions to this.

4 In this and the following sections, we drop the subscript *i* for simplicity.

5 On the other hand, it seems that the only way to generalize the conditioning argument to a model that also allows for time varying explanatory variables is to condition on equality of the explanatory variables across different time periods. Without such a restriction, the fixed effects in the denominators cannot cancel each other. Chountas and Kyriazidou (2021) pursue such a strategy for the conditional likelihood in a multinomial multivariate model with discrete explanatory variables. In the case of continuous explanatory variables, one may use the kernel weight approach introduced in Honoré and Kyriazidou (2000), although this would lead to an estimator that converges slower than the usual $\sqrt{n}$.

6 As was the case in Sect. 5.1, it seems that the only way to generalize the conditioning argument to a model that also allows for time varying variables is to condition on equality of the explanatory variables across different time periods.

7 The maximum score estimator in Manski (1987) can be motivated in terms of moment inequalities.

8 Subject to an identification condition that guarantees that the moment condition has a unique solution for $\rho$.

9 While the overall sample is large, each of the moment only depends on specific sequences that comprise very small fraction of the observations. In our application, these fractions ranged from less than 0.1% to 3%.

10 The p-value for the test is less than 1% in all cases. All test referred to in this paragraph are based on estimating $\exp (\rho )$ without imposing that it is positive, and then testing whether it differs from $\exp (0)$. The reason is that when we estimate $\rho$, we sometimes obtain a point estimate at the lower bound of the parameter space.
